# Inhibition of bromodomain and extra-terminal (BET) proteins increases NKG2D ligand MICA expression and sensitivity to NK cell-mediated cytotoxicity in multiple myeloma cells: role of cMYC-IRF4-miR-125b interplay

**DOI:** 10.1186/s13045-016-0362-2

**Published:** 2016-12-01

**Authors:** Maria Pia Abruzzese, Maria Teresa Bilotta, Cinzia Fionda, Alessandra Zingoni, Alessandra Soriani, Elisabetta Vulpis, Cristiana Borrelli, Beatrice Zitti, Maria Teresa Petrucci, Maria Rosaria Ricciardi, Rosa Molfetta, Rossella Paolini, Angela Santoni, Marco Cippitelli

**Affiliations:** 1 Department of Molecular Medicine - Pasteur Italia Laboratory, Sapienza University of Rome, Viale Regina Elena 291, 00161 Rome, Italy; 2 Center for Life Nano Science @ Sapienza, Italian Institute of Technology, Sapienza University of Rome, Rome, Italy; 3 Division of Hematology, Department of Cellular Biotechnologies and Hematology, Sapienza University of Rome, Rome, Italy; 4 Hematology, Department of Clinical and Molecular Medicine, Sapienza University of Rome, Rome, Italy; 5 Istituto Pasteur-Fondazione Cenci Bolognetti, Rome, Italy; 6 Istituto Mediterraneo di Neuroscienze Neuromed, Pozzilli, Italy

**Keywords:** Multiple myeloma, Bromodomain, IRF4, Natural killer, NKG2DLs

## Abstract

**Background:**

Anti-cancer immune responses may contribute to the control of tumors after conventional chemotherapy, and different observations have indicated that chemotherapeutic agents can induce immune responses resulting in cancer cell death and immune-stimulatory side effects. Increasing experimental and clinical evidence highlight the importance of natural killer (NK) cells in immune responses toward multiple myeloma (MM), and combination therapies able to enhance the activity of NK cells against MM are showing promise in treating this hematologic cancer. The epigenetic readers of acetylated histones bromodomain and extra-terminal (BET) proteins are critical regulators of gene expression. In cancer, they can upregulate transcription of key oncogenes such as cMYC, IRF4, and BCL-2. In addition, the activity of these proteins can regulate the expression of osteoclastogenic cytokines during cancer progression. Here, we investigated the effect of BET bromodomain protein inhibition, on the expression of NK cell-activating ligands in MM cells.

**Methods:**

Five MM cell lines [SKO-007(J3), U266, RPMI-8226, ARP-1, JJN3] and CD138^+^ MM cells isolated from MM patients were used to investigate the activity of BET bromodomain inhibitors (BETi) (JQ1 and I-BET151) and of the selective BRD4-degrader proteolysis targeting chimera (PROTAC) (ARV-825), on the expression and function of several NK cell-activating ligands (NKG2DLs and DNAM-1Ls), using flow cytometry, real-time PCR, transient transfections, and degranulation assays.

**Results:**

Our results indicate that inhibition of BET proteins via small molecule inhibitors or their degradation via a hetero-bifunctional PROTAC probe can enhance the expression of MICA, a ligand of the NKG2D receptor, in human MM cell lines and primary malignant plasma cells, rendering myeloma cells more efficient to activate NK cell degranulation. Noteworthy, similar results were obtained using selective CBP/EP300 bromodomain inhibition. Mechanistically, we found that BETi-mediated inhibition of cMYC correlates with the upregulation of miR-125b-5p and the downregulation of the cMYC/miR-125b-5p target gene IRF4, a transcriptional repressor of *MICA*.

**Conclusions:**

These findings provide new insights on the immuno-mediated antitumor activities of BETi and further elucidate the molecular mechanisms that regulate NK cell-activating ligand expression in MM.

**Electronic supplementary material:**

The online version of this article (doi:10.1186/s13045-016-0362-2) contains supplementary material, which is available to authorized users.

## Background

The epigenetic readers of acetylated histones, bromodomain and extra-terminal (BET) proteins, employ tandem bromodomains to recognize specific acetylated lysine residues in N-terminal tails of histone proteins. Members of the BET family including BRD2, BRD3, BRD4, and BRDT modulate gene expression, by recruiting transcriptional factors and chromatin-regulating enzymes to specific genomic locations [1]. Among the four known members of the BET proteins, BRD4 has been studied and characterized more in detail in recent years as a transcriptional coactivator of many cellular genes and in various regulatory pathways connected to different pathologies. Through its ability to recruit the elongation factor P-TEFb (a heterodimer of CDK9 and cyclin T) to the transcription start sites, it can induce the phosphorylation of the RNA polymerase II, activating the elongation of transcription [1, 2].

Under normal conditions, BET family proteins perform transcription regulatory functions, while in cancer, they can upregulate aberrant transcription of key oncogenes such as cMYC, BCL-2, and IRF4. Interestingly, many of these oncogenic drivers are regulated by a particular class of regulatory regions called super enhancers, formed by binding of high levels of specific transcriptional factors and coactivators (e.g., BRD4 and mediator) [3]. It has been proposed that genes regulated by super enhancers are particularly sensitive to BET inhibition [2], as demonstrated by genomewide analyses performed in MM1.S multiple myeloma (MM) cells and subsequently in Ly1 lymphoma cells [4]. In this context, oncogenes regulated by super enhancers can represent “druggable” targets for BET inhibitor-directed therapies.

The recent discovery (2010) of potent and highly specific small molecule inhibitors for the BET family of bromodomains (BETi), such as the triazolodiazepine-based JQ1 and the quinolone-based BET protein inhibitor I-BET151 among others, has shown that these molecules are able to bind to the KAc binding pocket of the bromodomains and disrupt their interactions with histones, thereby displacing BET proteins and their associated transcriptional regulatory complexes from chromatin (reviewed in [5]).

In this context, compelling preclinical evidence of BETi-mediated antitumor efficacy in refractory hematological malignancies has been provided, particularly in acute leukemia, myeloma, and some lymphomas, at drug levels that are achievable in vivo and with sufficient data to suggest acceptable off-target effects. Accordingly, these studies have led to the introduction of a number of early-phase, dose-escalation safety studies in the clinical arena, and several phase I trials using different BET inhibitor compounds covering most hematologic malignancies (including MM) are currently underway [6, 7] (https://clinicaltrials.gov/ct2/results?term=bromodomain+inhibitor&Search=Search).

MM is an incurable hematologic cancer characterized by clonal expansion of cancerous plasma cells in the bone marrow (BM) and its development is supported by a progressive impairment of immunosurveillance, mainly attributable to T lymphocyte and natural killer (NK) cell alterations [8]. Its development and progression is driven by transcriptional regulatory events that affect the differentiation of B cells to plasma cells, which subsequently support the growth of dysfunctional plasma cells. Deregulated activity of different transcription factors (TFs) has been implicated in MM development, including cMYC, MAF, NF-κB, and IRF4. The aberrant activity of these TFs in MM is demonstrated by the presence of translocation events that fuse them to active enhancers driving dysregulated expression. In this context, IRF4, a critical regulator of the normal adaptive immune response, plays a major role in MM progression; indeed, interference with IRF4 expression is lethal for these cells, irrespective of their genetic etiology, making IRF4 an “Achilles’ heel” that may be exploited therapeutically [9, 10]. Downstream targets of IRF4 include regulators of cell cycle progression, survival, and normal plasma cell function [9]. Interestingly, while oncogenic translocations of *IRF4* have been found, myeloma and other lymphoid malignancies are more frequently dependent on dysfunctional transcriptional networks downstream of a genetically normal *IRF4* locus [9].

NK cells are cytotoxic innate immune effectors involved in anti-cancer immune response, due to their ability to expand during the early stages of this disease and to recognize and lyse cancer cells. A number of evidence in myeloma patients strongly support the antitumor potential of NK cells in response to immunomodulatory drugs or following allogeneic stem cell transplantation [11–14]. In this regard, evidence is accumulating that the engagement of NKG2D and DNAM-1/CD226 activating receptors is critical for NK cell-mediated killing of MM, which express NKG2D and DNAM-1/CD226 ligands [8, 14–17]. However, BM and peripheral NK cells become unable to efficiently counteract MM as the disease progresses. Indeed, MM can inhibit NK cell functions directly, by producing immune suppressive factors and/or reducing their susceptibility to NK cell recognition. In addition, MM cells can undergo decreased surface expression of NK cell-activating ligands (e.g., NKG2DLs) [18], while expressing (together other cell population in the BM) ligands of inhibitory receptors such as the ligand of PD-1 (PD-L1) [19, 20], likely providing a mechanism of tumor escape.

Thus, improving NK cell responsiveness may be a promising therapeutic approach to treat MM; in particular, the modulation of the balance between activating and inhibitory NK cell signals and the sensitization of cancer cells to NK cell-mediated cytotoxicity may significantly contribute to enhance anti-myeloma immune responses.

We have previously defined several regulatory mechanisms of NK cell-activating ligand gene expression in MM cells [21] and recently demonstrated that immunomodulatory drugs (IMiDs—e.g., lenalidomide or pomalidomide) can upregulate cell surface expression of the activating ligands MICA and PVR/CD155 on MM, enhancing NK cell recognition and killing [13]. A prominent role in these regulatory mechanisms is played by the TFs IKZF1/3 and IRF4, able to repress the basal transcription of these genes. Thus, we identified IKZF1/3 and IRF4 as “druggable” transcriptional repressors of NK cell-activating ligand expression in MM, underlying the concept that targeting specific TFs critical for MM development and progression can cooperate at the same time with the activation of killer lymphocytes able to fight this cancer.

In this work, we describe the ability of BETi to upregulate the NKG2DL MICA (cell surface, messenger RNA (mRNA) expression and promoter activity) in MM cells, with little or no effects on the expression of other NKG2DL (e.g., MICB) and the DNAM-1L PVR/CD155. Moreover, exposure to BETi renders myeloma cells more efficient to activate NK cell degranulation.

Mechanistically, we found that BETi-mediated inhibition of cMYC expression correlates with the downregulation of its direct transcriptional target *IRF4* and with the upregulation of the microRNA-125b-5p (miR-125b-5p), a modulator of *IRF4* expression [22, 23]. Accordingly, lentiviral-mediated overexpression of miR-125b-5p inhibits IRF4 and increases MICA expression in MM cells, extending the possible immunoregulatory role of miR-125b-5p as promising anti-MM effector.

Finally, selective CREB-binding protein/E1A interacting protein of 300 kDa (CBP/EP300) bromodomain inhibition, already shown to affect MM viability through transcriptional suppression of IRF4 [24], recapitulated the observations obtained using pan-BETi on MICA expression, adding novel information on the possible immuno-therapeutic applications of this class of bromodomain inhibitors and transcriptional coactivators in MM.

In conclusion, these findings provide new insights on the immuno-mediated antitumor activities of BETi and further elucidate the molecular mechanisms that regulate NK cell-activating ligand expression in MM.

## Results

### BETi upregulate MICA expression on human multiple myeloma cells and enhance their recognition by NK cells

**Fig. 1:**
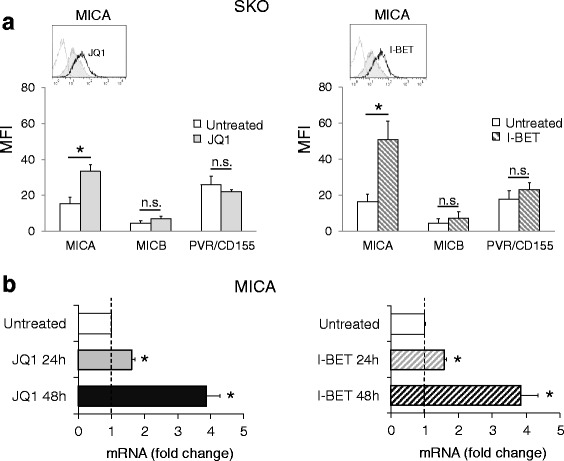
BETi upregulate MICA expression in SKO-007(J3) human MM cells. **a** MICA, MICB, and PVR/CD155 cell surface expression were analyzed by flow cytometry on SKO-007(J3) cells treated with JQ1 (0.5 μM) or I-BET151 (0.5 μM) for 72 h. Histograms represent MFI of specific mAb—MFI of isotype control. The MFI of MICA, MICB, and PVR/CD155 was calculated based on at least four independent experiments and evaluated by paired Student *t* test (*P < 0.05). In the *insert*, a representative histogram of MICA upregulation is shown. The *grey-colored* histograms represent basal expression of the indicated ligand, while *thick black* histograms represent the expression after treatment with the drug. **b** Real-time PCR analysis of total mRNA obtained from SKO-007(J3) cells, untreated or treated with the indicated BETi as described above for 24 and 48 h. Data, expressed as fold change units, were normalized with GAPDH and referred to the untreated cells considered as calibrator and represent the mean of three experiments (*P < 0.05)

We investigated the ability of BETi to regulate the expression of NK cell-activating ligands in MM. To this purpose, a panel of human MM cell lines [SKO-007(J3), RPMI-8226, U266, ARP-1, and JJN3] were treated with a range of concentrations and at different times with JQ1 or I-BET151, two small molecule bromodomain inhibitors displaying potent binding affinity to BET family proteins [25], and analyzed for the expression of NKG2DLs and DNAM-1Ls, by FACS analysis and qRT-PCR. As shown in Fig. 1a (and Additional file 1), these drugs upregulated the basal expression of the NKG2D ligand MICA in different MM cell lines, with no significant effects on MICB and PVR/CD155 levels. Concerning the other NKG2D and DNAM-1/CD226 ligands, SKO-007(J3) cells express low or undetectable levels of ULBP2/5/6 or ULBP1, ULBP3, and Nec-2 respectively and the treatment with BETi did not modify their cell surface levels (Additional file 2). These treatments did not affect the cell viability of these cell lines after 72 h treatment, as assessed by PI staining (data not shown).

**Fig. 2:**
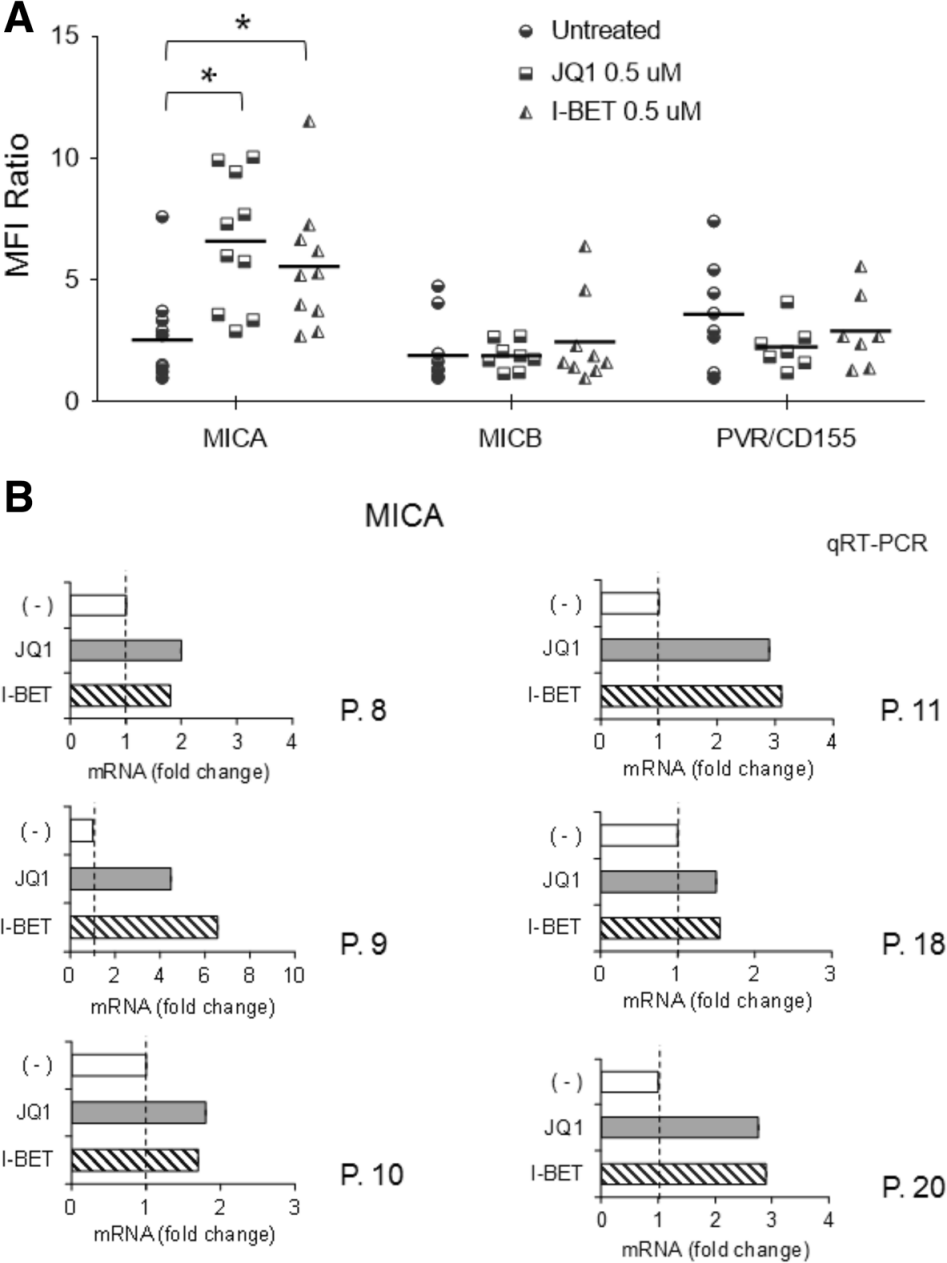
Upregulation of MICA expression on patient-derived PCs upon treatment with BETi. **a** MICA, MICB, and PVR/CD155 cell surface expression was analyzed by flow cytometry on patient-derived MM cells treated with the indicated BETi for 72 h. For each treatment, value ratio between MFI of specific mAb and MFI of isotype control is reported (*P < 0.05). **b** Real-time PCR analysis of total mRNA obtained from purified CD138^+^ cells untreated or treated with JQ1 or I-BET151 for 48 h in complete medium supplemented with 20 ng/ml IL-3 and 2 ng/ml IL-6. Data, expressed as fold change units, were normalized with GAPDH and referred to the untreated cells considered as calibrator. Myeloma cells were selected using anti-CD138 magnetic beads and more than 95% of the purified cells expressed CD138 and CD38

To examine whether the upregulation of MICA by BETi could be associated with increased mRNA levels, total RNA was isolated from the different cell lines exposed to JQ1 or I-BET151 and analyzed by real-time qRT-PCR. As shown in Fig. 1b and Additional file 3, we noticed a significant increase of MICA mRNA levels in treated cells, in particular after 48 h. In addition, we confirmed these results also in CD138^+^ MM cells isolated from the bone marrow of MM patients (Table 1), showing higher cell surface and mRNA levels of MICA following treatment with JQ1 or I-BET151 (Fig. 2a, b). Of note, these drugs did not show a significant effect on either MICB or PVR/CD155, independently from the clinical status of the disease and from basal expression levels.

**Table 1:** Clinical parameters of MM patients

Patient	Sex/age	Clinical state	Monoclonal Ig	% PCs in BM
1	F/42	Onset	IgG-k	52
2	M/71	Relapse	IgG-L	29
3	M/52	Onset	IgA-k	30
4	M/73	Smoldering	IgG-L	22
5	F/73	Smoldering	IgA-L	34
6	M/50	Onset	IgG-L	60
7	F/68	Relapse	IgG-L	19
8	M/75	Relapse	Micro-k	90
9	M/58	Onset	IgG-k	60
10	F/46	Onset	IgG-L	67
11	M/75	Relapse	IgG-k	32
12	M/71	Relapse	IgG-k	52
13	F/78	Relapse	IgG-k	51
14	M/68	Relapse	IgG-k	44
15	M/75	Onset	IgG-L	24
16	F/54	Onset	IgG-L	34
17	F/61	Relapse	IgG-L	38
18	F/78	Onset	IgG-k	40
19	M/73	Smoldering	IgG-λ	13
20	M/71	Onset	IgG-k	60

Patients were classified according to the status of disease. The percentage of plasma cells in the BM and monoclonal Ig is indicated

**Fig. 3:**
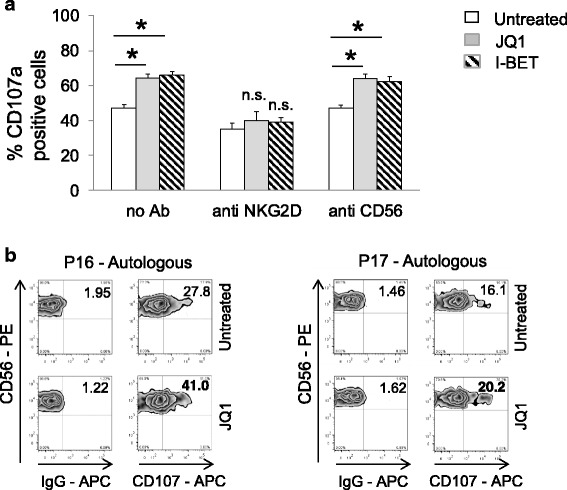
BETi increase susceptibility of MM cells to NK cell recognition and degranulation. **a** NK cells, prepared from PBMCs of healthy donors, were incubated with SKO-007(J3) cells, untreated or treated with the indicated BETi for 72 h as described above, and used as target cells in a degranulation assay. The assay was performed at the effector/target (E:T) ratio of 2.5:1. After 2 h at 37 °C, cells were stained with anti-CD56, anti-CD3, and anti-CD107a mAbs. Cell surface expression of CD107a was analyzed on FSC/SSC-gated and CD56^+^CD3^−^ cells. In order to evaluate the role of NKG2D, the assay was performed in parallel treating NK cells with a blocking anti-NKG2D antibody. Percentage of CD107a positive cells was calculated based on five independent experiments and evaluated by paired Student *t* test (*P < 0.05). **b** BETi increase susceptibility of patient-derived MM PCs cells to autologous NK cell recognition and killing. CD138^−^ bone marrow cells, cultured for 2 days in complete medium supplemented with IL-2 (200 U/mL), were incubated with purified autologous myeloma cells, untreated or treated with JQ1 for 48 h, and used as target cells in a degranulation assay. The assay was performed at the effector/target (E:T) ratio of 2.5:1. After 2 h at 37 °C, cells were stained with anti-CD56, anti-CD3, anti-CD16, and anti-CD107a mAbs. Cell surface expression of CD107a was analyzed on CD56^+^CD16^+^CD3^−^ cells. Results obtained from two patients (P16 and P17) are represented

To investigate the functional consequences of BETi-induced changes of MICA expression, we analyzed the lysosomal marker CD107a (a surrogate marker for granule mobilization [26]) on NK cells isolated from healthy donors against SKO-007(J3) cells, untreated or treated with JQ1 or I-BET151, by FACS analysis. As shown in Fig. 3a, basal expression of CD107a on NK cells was enhanced when co-cultured with SKO-007(J3) target cells exposed to BETi; this effect was significantly inhibited by a blocking anti-NKG2D monoclonal antibody (mAb), indicating that stimulation of NK cell degranulation was dependent on NKG2D activation. Accordingly, a higher capability of degranulation was also observed in patient-derived NK cells against BETi-treated autologous MM targets (Fig. 3b).

Altogether, these data indicate that treatment of human MM cells with BETi enhances MICA cell surface and mRNA expression, increasing their susceptibility to NK cell recognition and killing.

### BETi-induced upregulation of MICA in MM cells: transcriptional mechanisms

**Fig. 4:**
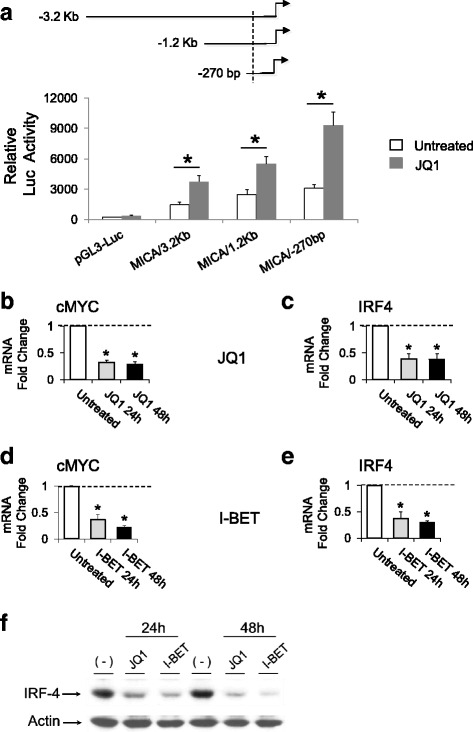
*MICA* promoter activity is enhanced by BETi in MM cells. **a** SKO-007(J3) cells were cotransfected with 3 μg of the indicated luciferase reporter vector + 1 μg of pRL-TK expression vector as described in the “Methods” section. Four hours after transfection, cells were left untreated (−) or were stimulated with 0.5 μM JQ1 for 48 h. Cells were then harvested and protein extracts were prepared for the luciferase and Renilla assays. Results are expressed as relative luciferase activity normalized to protein concentration as well as to Renilla activity produced off the internal control plasmid and represent the mean value of four independent experiments (*P < 0.05). **b–e** BETi represses cMYC and IRF4 mRNA expression in SKO-007(J3) MM cells. Real-time PCR analysis of total mRNA obtained from SKO-007(J3) cells, untreated or treated with the indicated BETi as described above for 24 and 48 h. Data, expressed as fold change units, were normalized with GAPDH and referred to the untreated cells considered as calibrator and represent the mean of three experiments (*P < 0.05). **f** Lysates of SKO-007(J3) cells untreated or treated with BETi for 24 or 48 h were subjected to Western blotting using anti-IRF4 and actin antibodies. The proteins transferred to nitrocellulose membranes were stained with Ponceau to verify that similar amounts of proteins had been loaded in each lane. Data are representative of one out of three independent experiments

To explore the possibility that BETi could increase MICA expression at the transcriptional level, we analyzed the effects of BET proteins inhibition on SKO-007(J3) cells transiently transfected with different MICA 5′-flank promoter fragments, cloned upstream of a luciferase reporter gene. As shown in Fig. 4a, JQ1 enhances the activity of progressive promoter deletions (−3.2 kb to −270 bp from the translation start site), delimiting a minimal fragment spanning from −270 bp still responsive to BETi.

Downregulation of cMYC and of cMYC-dependent signatures in MM cells (e.g., IRF4 and IRF4-dependent oncogenic targets [2, 23]) have been proposed as pivotal effectors of BETi-mediated activity in this cancer. In this regard, our previous observation has identified IRF4 as an IMiD “druggable” transcriptional repressor of NK cell-activating ligands in MM cells [13], suggesting that similar transcriptional pathways could be involved in BETi-treated cells.

We focused our attention on the immune-regulatory interplay mediated by these BET protein-regulated TFs and the regulation of MICA expression in MM. As shown in Fig. 4b–f and Additional file 4, we confirmed the capability of BETi to modulate the expression of cMYC and IRF4 both in SKO-007(J3) and in primary plasma cells isolated from MM patients. In addition, compared with non-targeting short hairpin RNA (shRNA)-infected cells, cMYC shRNA-transduced cells expressed higher MICA surface levels, whereas MICB and PVR/CD155 membrane expression were unaffected (Additional file 5A). Accordingly, real-time qRT-PCR analysis showed that silencing of cMYC enhances MICA mRNA expression and represses IRF4 (Additional file 5B).

**Fig. 5:**
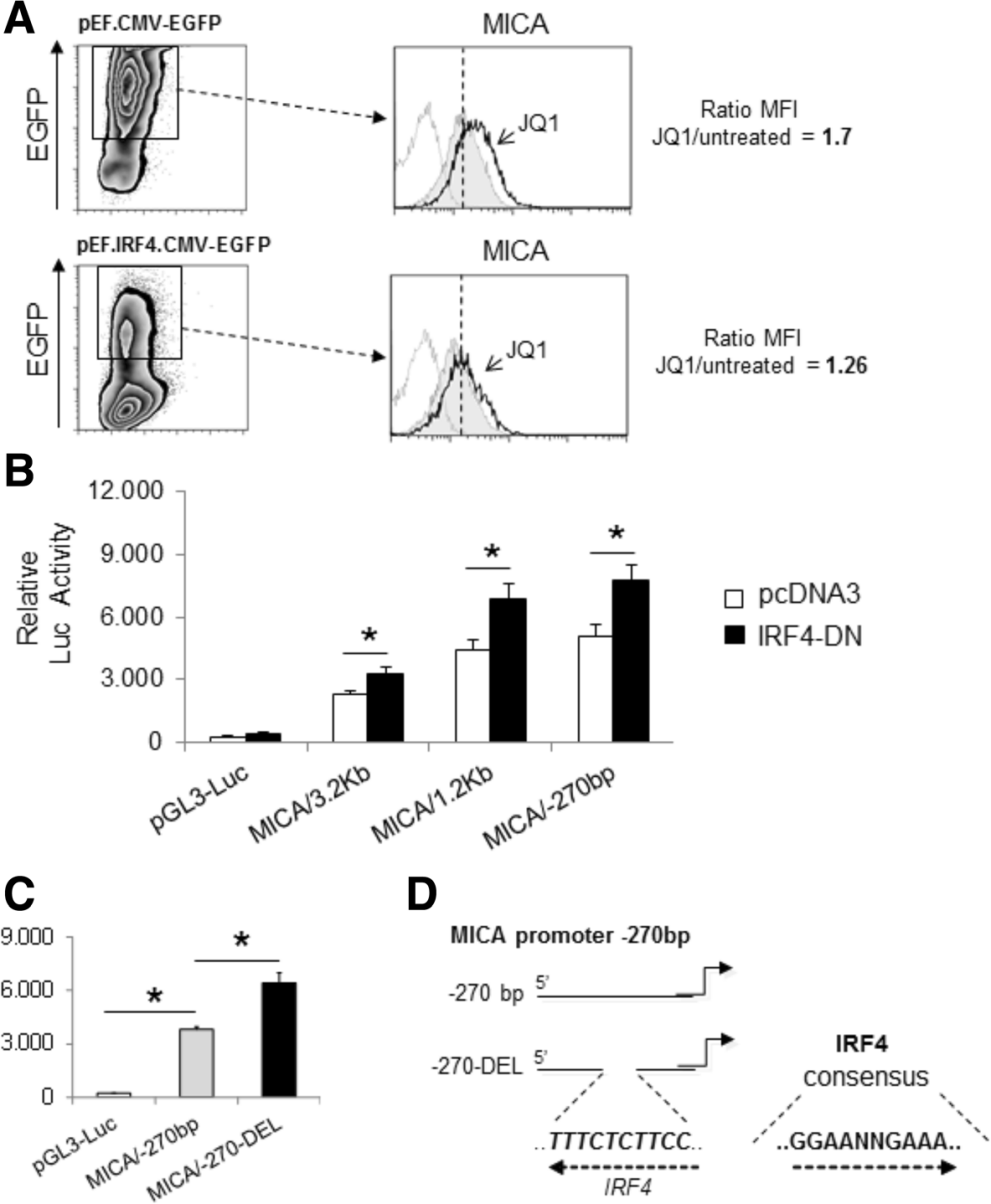
IRF4 is a repressor of *MICA* promoter activity in MM cells. **a** MICA cell surface expression was analyzed by flow cytometry on pEF.CMV.EGFP-IRF4-lentivirus (or pEF.CMV.EGFP control virus) infected SKO-007(J3) cells (72 h), by gating on GFP^+^ cells as indicated in the panel. After infection, cells were separated into different drug treatment groups, untreated or treated with JQ1 (0.5 μM). The ratio MFI between JQ1-treated on untreated cells is shown in a representative experiment. **b** SKO-007(J3) cells were cotransfected with 3 μg of the indicated luciferase reporter vector and pRL-TK as described above + 1 μg of an expression vector encoding a truncated form of the human IRF4, IRF4-DN, or an empty control vector pcDNA3. After 48 h, cells were harvested and protein extracts were prepared for the luciferase and Renilla assays. Results are expressed as relative luciferase activity normalized to protein concentration as well as to Renilla activity produced off the internal control plasmid and represent the mean value of four independent experiments (*P < 0.05). **c** SKO-007(J3) cells were transfected with 3 μg of the indicated luciferase reporter vector and pRL-TK as described above. After 48 h, cells were harvested and protein extracts were prepared for the luciferase and Renilla assays. Results are expressed as relative luciferase activity normalized to protein concentration as well as to Renilla activity produced off the internal control plasmid and represent the mean value of four independent experiments (*P < 0.05). **d** Schematic representation of the IRF4 response element deleted in the mutant promoter MICA/-270-DEL

IRF4 can positively and negatively regulate different genes in part by binding to distinct DNA binding motifs and through interactions with various additional transcription factors [27]. As shown in Fig. 5a, SKO-007(J3) cells transiently infected with a bicistronic lentivirus expressing IRF4 along with EGFP, displayed a significant lower induction of MICA expression in the presence of JQ1 as compared to cells infected with an empty control vector. Accordingly, the selective abrogation of IRF4 transcriptional activity affects *MICA* promoter in MM cells; as shown in Fig. 5b and consistent with our previous study [13], transient transfection of a truncated form of IRF4 (consisting of its N-terminal DNA binding domain IRF4-DN/1-405) was sufficient to increase MICA promoter activity, presumably by competing with and inhibiting the endogenous repressive activity of IRF4 on *MICA*. In addition, as shown in Fig. 5c, d and Additional file 6A, the deletion of a putative IRF4 binding region in the MICA/−270-bp fragment increased the transcriptional activity. Interestingly, the same binding region is not fully conserved in the *MICB* promoter, as already described for the contiguous Ikaros binding elements in the same region. Finally, as shown in Additional file 6B–D, treatment of SKO-007(J3) cells with JQ1 only slightly decreased the expression of the *MICA* repressors IKZF1 and IKZF3 [13] as compared to the severe degradation induced by lenalidomide (via cereblon-CRL4) [28, 29], suggesting that the upregulation of MICA was mainly dependent on IRF4 downregulation. Interestingly, the combined treatment JQ1 + lenalidomide further increased the expression of MICA and repressed IRF4, suggesting that cereblon-mediated degradation of IKZF1/3 cooperates with BETi-mediated repression of IRF4 in this context (Additional file 6E, F).

Thus, MICA mRNA expression and promoter activity are enhanced by BETi via a mechanism dependent on IRF4 downregulation.

### Cross-talk BETi/miR-125b/IRF4 and regulation of MICA expression in MM cells

Targeting deregulated microRNAs (miRNAs) is emerging as a novel promising therapeutic approach against a number of cancers, including MM. Indeed, replacement of tumor suppressor miRNAs by synthetic oligonucleotides (miRNA mimics) offers a new therapeutic opportunity to restore a loss of function or to regulate pathways involved in the control of cancer progression [30, 31].

The microRNA-125b-5p (miR-125b) is commonly deregulated in cancer, where its expression is generally repressed by the activity of cMYC [32]; however, its function diverges in different malignancies with dependence on the molecular contexts [32]. Noteworthy, recent findings have shown that the expression of IRF4 is modulated by miR-125b-5p in MM cell lines or in patient-derived MM cells, and enforced expression of miR-125b-5p can affect cell growth and survival via downregulation of IRF4 and impairment of its downstream signaling, indicating that miR-125b is a tumor suppressor in MM [23].

**Fig. 6:**
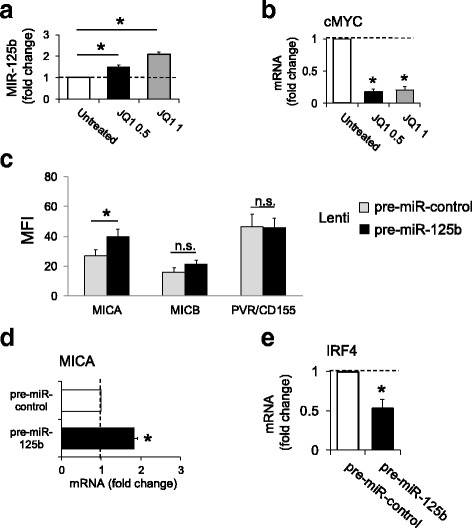
miR-125b upregulates MICA expression in SKO-007(J3) human MM cells. **a** Treatment with JQ1 (48 h) increases miR-125b expression in SKO-007(J3) cells. **b** As a control, the same mRNAs were analyzed for cMYC expression. Data of real-time PCR analysis, expressed as fold change units, were normalized with (U6) or GAPDH respectively and referred to the untreated cells considered as calibrator and represent the mean of three experiments (*P < 0.05). **c** Overexpression of pre-miR-125b increases MICA cell surface expression in SKO-007(J3) cells. MICA, MICB, and PVR/CD155 surface expression were analyzed by flow cytometry on pre-miR-control/GFP or pre-miR-125b/GFP lentivirus transiently infected SKO-007(J3) cells (72 h), by gating on GFP^+^ cells. The MFI of MICA, MICB, and PVR/CD155 was calculated based on at least four independent experiments and evaluated by paired Student *t* test (*P < 0.05). **d**, **e** Overexpression of pre-miR-125b increases MICA mRNA expression and represses IRF4 mRNA expression in SKO-007(J3) cells. Real-time PCR analysis of total mRNA obtained from SKO-007(J3) cells infected with pre-miR-control or pre-miR-125b lentivirus. Data, expressed as fold change units, were normalized with GAPDH and referred to the pre-miR-control infected cells considered as calibrator and represent the mean of three experiments (*P < 0.05)

In agreement with the studies mentioned above, our observations show that treatment of MM cells with BETi induces the upregulation of miR-125b-5p and this correlates with the inhibition of cMYC expression (Fig. 6a, b). In the same experimental setting, we confirmed the repression of IRF4 and the induction of MICA mRNA (Additional file 7A, B). Accordingly, lentiviral-mediated overexpression of this miRNA in SKO-007(J3) cells upregulates cell surface and mRNA levels of MICA (Fig. 6c, d) and inhibits the expression of IRF4 (Fig. 6e), highlighting an additional/overlapping miR-mediated immunoregulatory pathway for *MICA* expression associated with BET proteins inhibition in MM.

### BETi-induced upregulation of MICA in MM cells: role of BRD4

Recent studies have recognized the important role of the BET family member BRD4 at super enhancer regions involved in the regulation of specific oncogenes in different cancers, including MM [2, 4, 33–37], and highlighted the feasibility of blocking its function using BETi. However, a potential problem with the use of small molecule inhibitors is their reversible binding and significant systemic drug concentrations and/or continuous exposures are often required to ensure sufficient functional inhibition.

Recently, novel small molecule-induced protein degradation strategies have been proposed for targeting undruggable targets. In particular, the proteolysis targeting chimeras (PROTACs) are able to recruit targeted proteins to the E3 ubiquitin ligases for ubiquitination and subsequent proteasome-mediated degradation [38–41].

**Fig. 7:**
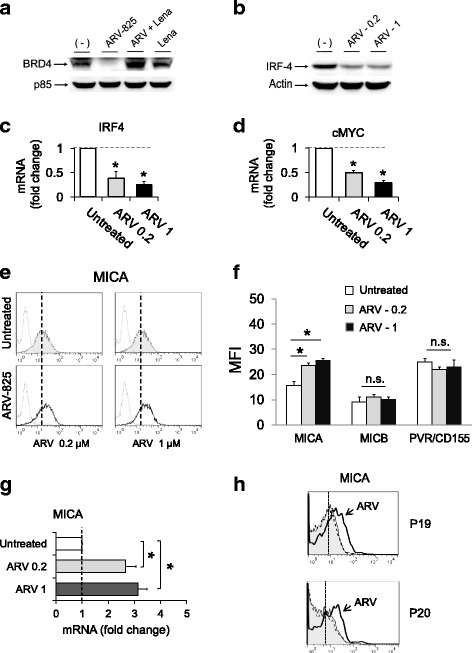
The PROTAC/BRD4 degrader, ARV-825, upregulates MICA expression in SKO-007(J3) cells. **a** Treatment of SKO-007(J3) cells with ARV-825 induced a strong degradation of BRD4 that was reverted in the presence of lenalidomide. Lysates of SKO-007(J3) cells, untreated or treated with ARV-825 (0.2 μM), lenalidomide (10 μM), or the combination of the two drugs for 24 h, were subjected to Western blotting using anti-BRD4 or anti-p85 antibodies. **b** ARV-825 inhibits the expression of IRF4. Lysates of SKO-007(J3) cells, untreated or treated with the indicated (μM) concentrations of ARV-825 for 48 h, were subjected to Western blotting using anti-IRF4 or anti-actin antibodies. The proteins transferred to nitrocellulose membranes were stained with Ponceau to verify that similar amounts of proteins had been loaded in each lane. Data are representative of one out of three independent experiments. **c**, **d** ARV-825 inhibits mRNA expression of IRF4 and cMYC. Real-time PCR analysis of total mRNA obtained from SKO-007(J3) cells, untreated or treated with the indicated (μM) concentrations of ARV-825 for 48 h. Data, expressed as fold change units, were normalized with GAPDH and referred to the untreated cells considered as calibrator and represent the mean of three experiments (*P < 0.05). **e**, **f** MICA, MICB, and PVR/155 cell surface expression were analyzed by flow cytometry on SKO-007(J3) cells treated with the indicated concentrations of ARV-825 for 72 h. The MFI of MICA, MICB, and PVR/155 were calculated based on at least four independent experiments and evaluated by paired Student *t* test (*P < 0.05). In **e**, a representative histogram of MICA upregulation is shown. The *grey-colored* histograms represent basal expression of the indicated ligand, while *thick black* histograms represent the expression after treatment with the drug. Data are representative of one out of four independent experiments. **g** ARV-825 increases mRNA expression of MICA in SKO-007(J3) cells. Real-time PCR analysis of total mRNA obtained from SKO-007(J3) cells, untreated or treated with the indicated (μM) concentrations of ARV-825 for 48 h. Data, expressed as fold change units, were normalized with GAPDH and referred to the untreated cells considered as calibrator and represent the mean of three experiments (*P < 0.05). **h** MICA cell surface expression was analyzed by flow cytometry on patient-derived MM cells treated with the indicated ARV-825 for 72 h. The *grey-colored* histograms represent basal expression, while *thick black* histograms represent the expression after treatment with the drug

In order to better investigate the role of BRD4 in BETi-mediated upregulation of MICA and the possibility to target its function using a protein degradation strategy, we treated MM cells with ARV-825, a novel PROTAC consisting of a classic BRD4 binding moiety (triazolodiazepine acetamide class in the BETi OTX015) and the compound pomalidomide, a third-generation immunomodulatory drug able to bind to the E3 ubiquitin ligase cereblon [42] connected by a flexible polyethylene glycol linker (Additional file 8). With this structure, ARV-825 has the ability to recruit BRD4 to cereblon, resulting in the rapid and efficient degradation of the former via the proteasome [43]. As shown in Fig. 7a, treatment of SKO-007(J3) cells with ARV-825 induced a strong degradation of BRD4 that was reverted in the presence of lenalidomide (a competitor for binding to cereblon). In the same experimental setting, ARV-825 significantly downregulated the expression of IRF4 and cMYC (Fig. 7b–d) and upregulated the expression of MICA (cell surface and mRNA), similarly to what observed in the presence of JQ1 or I-BET151 (Fig. 7e–g). We confirmed these results also in CD138^+^ MM cells isolated from the bone marrow of MM patients, showing higher cell surface levels of MICA following treatment with ARV-825 (Fig. 7h).

Moreover, the role of BRD4 was further confirmed by shRNA interference (Additional file 9A, B). Since stable BRD4 depletion is cytotoxic for myeloma cells, in these experiments were used transient infections. Compared with non-targeting shRNA-infected cells, BRD4 shRNA-transduced cells expressed higher MICA cell surface levels.

### Enhancing bromodomain selectivity: MICA expression is upregulated by bromodomain inhibition of the transcriptional coactivators EP300/CBP in MM cells

Preclinical evidence of the antitumor efficacy of BETi in refractory hematological malignancies, including MM, investigated the action of drug levels that are achievable in vivo, with sufficient data showing acceptable toxicity. However, significant off-target effects have been described, rising concern on the safety consequences of BET inhibition.

Given the broad involvement of BET proteins in transcriptional regulation, improvement of the selectivity of these compounds would restrict the number of genes potentially affected, increasing specific targeting to avoid adverse effects in the clinic [44].

In the last few years, selective and highly potent chemical probe compounds targeting the bromodomains of CBP/EP300 (CBP/EP300-BRi) have been developed (e.g., SGC-CBP30 and I-CBP112), with high selective affinity for the bromodomains of CBP/EP300 over other bromodomains (greater than 30-fold selectivity of CBP30 for CBP and EP300 compared with other bromodomains) [24].

CBP and EP300 are highly homologous bromodomain-containing transcriptional coactivators that regulate a number of important cellular events through their acetyltransferase activity. Genetic studies in mice and analysis of human cancer mutations and translocations have implicated CBP/EP300 in cancer, but the role of the bromodomain in the normal and pathological function of CBP/EP300 has not been extensively studied.

Noteworthy, CBP/EP300 bromodomain inhibition has been shown to target a more limited subset of hematologic cell lines, with a bias toward MM/plasmacytoma cell lines and enrichment of signatures for pathways important in MM growth and survival. This increased selectivity was distinct from the effects of pan-BET inhibition, targeting the IRF4-MYC network at different nodes and, interestingly, via the direct focused transcriptional inhibition of IRF4 [24].

**Fig. 8:**
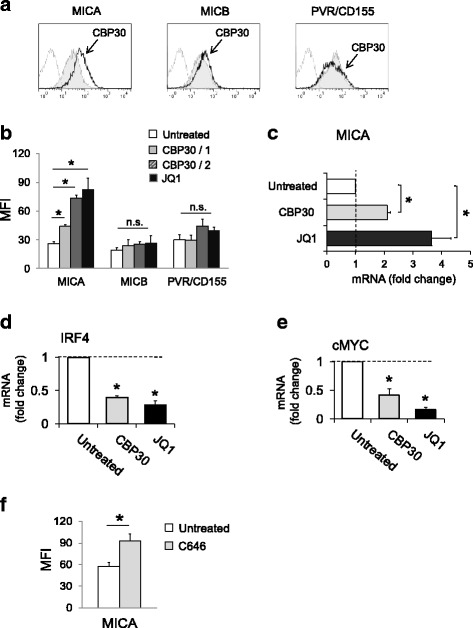
CBP/EP300-BRi upregulate MICA expression in SKO-007(J3) MM cells. **a**, **b** MICA, MICB, and PVR/CD155 cell surface expression were analyzed by flow cytometry on SKO-007(J3) cells treated with CBP30 (1 or 2 μM) for 72 h. In **a**, a representative histogram of MICA upregulation compared to MICB and PVR/CD155 is shown (2 μM). The MFI of MICA, MICB, and PVR/CD155 was calculated based on at least four independent experiments and evaluated by paired Student *t* test (*P < 0.05). **c** Real-time PCR analysis of total mRNA obtained from SKO-007(J3) cells, untreated or treated with CBP30 or JQ1 as described above for 48 h. Data, expressed as fold change units, were normalized with GAPDH and referred to the untreated cells considered as calibrator and represent the mean of three experiments (*P < 0.05). **d**, **e** CBP/EP300-BRi inhibits mRNA expression of IRF4 and cMYC. Real-time PCR analysis of total mRNA obtained from SKO-007(J3) cells, untreated or treated with CBP30 or JQ1 as described above, for 48 h. Data, expressed as fold change units, were normalized with GAPDH and referred to the untreated cells considered as calibrator and represent the mean of three experiments (*P < 0.05). **f** MICA cell surface expression was analyzed by flow cytometry on SKO-007(J3) cells treated with C646 (5 μM) for 72 h. The MFI of MICA was calculated based on at least three independent experiments and evaluated by paired Student *t* test (*P < 0.05)

Our observations indicate that treatment of human MM cell lines with CBP/EP300-BRi (SGC-CBP30) (24 to 72 h) selectively upregulates cell surface and mRNA expression of MICA with no significant effects on MICB or PVR/CD155 (Fig. 8a–c), suggesting an immunomodulatory potential associated with the specific CBP/EP300 bromodomain inhibition. In agreement with the above-described data, SCG-CBP30 repressed the expression of IRF4 and cMYC in our experimental system, although to a lesser extent than JQ1 (Fig. 8d, e), suggesting that significant upregulation of MICA can be achieved in MM cells also in the presence of inhibitors able to affect the function of selected bromodomains, specifically involved in the control of the IRF4-regulated transcriptional network. Interestingly, as shown in Fig. 8f, treatment of SKO-007(J3) cells with a specific-competitive histone acetyltransferase CBP/EP300 inhibitor (C646) [45, 46] was able to replicate a similar upregulation of MICA in our experimental system, thus identifying CBP/EP300 as novel regulators of this gene in MM.

## Discussion

In this study, we investigated the effects of BETi on NK cell-activating ligand expression in MM cells.

The data shown in this manuscript indicate that the mRNA and cell surface expression of the NK cell-activating ligand MICA is upregulated in BETi-treated human MM cells lines and in MM cells isolated from the bone marrow of MM patients, independently from the clinical stage of the disease and from basal level of expression of this ligand. The functional implication of this upregulation is an increased degranulation of NK cells contacting drug-treated target cells, with a mechanism dependent on the activation of the NKG2D receptor, either using cultivated NK cells from healthy donors or autologous patient-derived NK cells (Fig. 3).

Mechanistically, we found that treatment of MM cells with BETi upregulates the activity of the *MICA* promoter; moreover, using progressive deletions, we identified a minimal promoter fragment spanning from −270 bp still responsive to BETi (Fig. 4).

Treatment of MM cells with BETi downregulated the expression of cMYC and IRF4 (Fig. 4 and Additional file 4), the latter being a transcriptional target of cMYC, and recently identified by our laboratory as a “IMiDs druggable” transcriptional repressor of MICA in MM cells [13]. In this context, cMYC shRNA-transduced cells expressed higher MICA surface levels, whereas MICB and PVR/CD155 membrane expression were unaffected. Accordingly, real-time qRT-PCR analysis showed that silencing of cMYC enhances MICA mRNA expression and represses IRF4 (Additional file 5).

IRF4 can positively and negatively regulate different genes, in part by binding to distinct DNA binding motifs and through interaction with various additional transcription factors [27]. Its C-terminal transactivation domain is critical for gene activation, while the mechanism(s) responsible for gene repression are not well defined. In our experimental system, the overexpression of IRF4 by lentiviral-mediated transduction partially abrogated the induction of MICA after treatment with BETi. Moreover, the overexpression of a dominant negative form of IRF4 induced significant upregulation of *MICA* promoter, and the deletion of a putative IRF4 binding element in the minimal MICA/-270 bp promoter fragment enhanced its activity, further confirming the repressive activity of IRF4 at this level (Fig. 5).

Interestingly, the expression of IRF4 has been linked to the activity of the transcription factors IKZF1 and IKZF3 [28, 29, 47], two recently identified repressors of *MICA* gene expression [13]. However, in our experimental system, treatment of SKO-007(J3) cells with JQ1 only slightly decreased the expression of the IKZF1 and IKZF3 (Additional file 6A–C); as a control, lenalidomide completely abrogated the expression of these two transcription factors, further suggesting that the upregulation of MICA mediated by BETi was mainly dependent on the repression of IRF4. Noteworthy, as shown in Additional file 6E, the combination of the two treatments (lenalidomide + BETi) further increased the expression of MICA, suggesting the possibility that low-dose combinations of IMiDs with BETi could display additional or synergistic activities in MM, as already shown in primary effusion lymphoma (PEL) preclinical data, where the simultaneous targeting of IKZF1-IRF4-MYC increased the cytotoxic effect as compared with either agent alone [48]. In this context, in MM, the combined activities of these two classes of drugs could be able to improve direct cytotoxicity and, at the same time, to enhance the expression of NK cell-activating ligands. Further experiments will be needed to better investigate this possibility.

Several reports have shown that different cellular miRNAs regulate NKG2DL genes, and the expression of several miRNAs that target NKG2D ligands is regulated by immune stimuli or activated p53, suggesting a complex regulatory role [49–51]. In MM, miRNA dysregulation has been shown to increase from MGUS to MM patients and specific miRNA signatures were determined [52, 53]. In this regard, recent findings revealed a peculiar role played by miR-125b in MM. Differently from other hematologic malignancies, this miRNA displays tumor suppressor activities in MM, affecting growth and survival, impairing the expression of IRF4 and its downstream targets [23]. In our experimental system, treatment of MM cells with BETi induced upregulation of miR-125b-5p and this correlated with downregulation of cMYC, as already described in different malignancies [54]. Lentiviral-mediated overexpression of this miR-inhibited the expression of IRF4 and upregulated cell surface and mRNA levels of MICA (Fig. 6), suggesting an additional miR-mediated immune-regulatory network associated with BET inhibition, cMYC, IRF4, and MICA expression. Interestingly, recent findings indicate that synthetic miR-125b-5p mimics can induce anti-myeloma activity in vivo, with no significant toxic effects (e.g., PBMC viability), suggesting a promising therapeutic activity [23]. Further experiments will be needed to investigate the possibility to extend this therapeutic strategy to harness the cytotoxic activity of NK cells against MM via MICA/NKG2D.

BETi are currently being evaluated in different clinical trials for a range of malignancies, due to their ability to suppress the expression of otherwise undruggable downstream transcription factors; however, the molecular and cellular mechanisms that regulate sensitivity or the resistance to these drugs remain largely unknown and the identification of relevant predictive biomarkers of response is needed [55].

Among the potential problems connected with the use of BETi in therapy is the finding that selected drugs (e.g., JQ1 or OTX015) have been shown to induce significant accumulation of BRD4 protein in Burkitt’s lymphoma cell lines; in addition, similar observations have been reported in lung and prostate cancer cell lines [43, 56]. It has been suggested that binding of BETi to BRD4 could result in a conformational change leading to increased stability of this protein and/or altered BRD4 accessibility to the endogenous cellular degradation machinery. Moreover, this increase of BRD4 levels and the reversible nature of inhibitor binding could preclude efficient BRD4 inhibition and repression of cMYC [43], thus limiting the potential benefit of therapeutic intervention at clinically achievable concentrations of these inhibitors.

Our data indicate that treatment of SKO-007(J3) cells with the PROTAC BRD4-degrader ARV-825 (sub-micromolar concentrations) induced a strong cereblon-mediated degradation of BRD4 with significant downregulation of IRF4-cMYC and upregulation of MICA. In this context, the role of BRD4 was further confirmed by direct shRNA interference (Additional file 9). Thus, BRD4-PROTACs could represent an additional promising strategy to efficiently block the BRD4/cMYC/IRF4 axis, increasing at the same time the expression of MICA in MM.

A different problem concerning the use of BETi in therapy could be related to off-target effects that they can induce. For example, the pan-BET inhibitory activity of JQ1 has been shown to inhibit testicular BRDT to cause reversible testicular atrophy and infertility [57], and thrombocytopenia together neutropenia were the dose-limiting toxicities observed in phase I clinical trials with the oral BET-BRD inhibitor OTX015 [6, 7]. Noteworthy, the proposed/tolerated dose of this inhibitor (40 to 80 mg once daily) has been shown to reach plasma drug levels in the micromolar range [6, 7], a concentration able to induce a significant upregulation of MICA expression in our experimental setting (Additional file 10).

Thus, improvement of BETi activity toward selected members of this family of regulators would restrict the number of genes potentially affected, improving specific targeting [44].

In this regard, specific inhibition of CBP/EP300 bromodomains has been shown to target a more limited subset of hematological malignant cells, with a bias toward multiple myeloma/plasmacytoma cell lines. This increased selectivity was distinct from the effects of pan-BET inhibition, targeting the IRF4-cMYC network at different nodes and, interestingly, via direct focused transcriptional inhibition of IRF4 [24]. Our observations indicate that treatment of MM cells with micromolar concentrations of a CBP/EP300-BRi (SGC-CBP30) repressed the expression of IRF4 and upregulated cell surface and mRNA expression of MICA (Fig. 8), suggesting an immunomodulatory potential associated with the selective inhibition of the CBP/EP300 bromodomain. The higher affinity for the bromodomains of CBP/EP300 over other bromodomains (greater than 30-fold selectivity of CBP30 for CBP/EP300 compared with other bromodomains) is predicted to restrict possible unwanted off-target effects [24, 58].

Interestingly, a distinct association between abnormal EP300 and different malignancies has been characterized in the last years [59–62]. Indeed, in addition to growth arrest, EP300 is involved in G1/S transition in human cancer cells and its inhibition can induce block of progression to the S-phase and apoptosis [45, 61]. The finding reported here that a specific histone acetyltransferase CBP/EP300 inhibitor (C646) is able to replicate a similar upregulation of MICA (at least in our experimental system), identifies CBP/EP300 as possible novel and druggable regulator(s) of this gene in MM, via specific classes of small molecule probes. Further experiments will be needed to better investigate the role of CBP/EP300 as a regulator of *MICA* gene expression in patient-derived MM cells and in other hematological tumors.

## Conclusions

In summary, we propose a model in which the functional inhibition of BET proteins or CBP/EP300 using specific inhibitors can increase the expression of the NKG2D-activating ligand MICA in MM cells via different integrated pathways involving the axis cMYC-IRF4-miR-125b.

These data add novel information on the immunomodulatory activities displayed by BETi and CBP/EP300-BRi, small molecules already known to modulate the expression of inflammatory cytokines in autoimmunity, to inhibit deregulated osteoclastogenesis during cancer progression and to block Th17 and Treg function, two important T cell subsets in MM pathobiology [58, 63–67].

The possibility to obtain a much more restricted/tailored effect on gene expression and immunomodulation using novel BETi or CBP/EP300-BRi [58] could further improve direct and immuno-mediated antitumor activity of bromodomain inhibition in MM and add novel information on the molecular mechanisms that regulate NK cell-activating ligand expression.

## Methods

### Cell lines and clinical samples

Human myeloma cell lines SKO-007(J3), U266, ARP-1, and RPMI-8226 were kindly provided by Prof. P. Trivedi (University of Rome, Sapienza, Italy). The human MM cell lines JJN-3 was kindly provided by Prof. N. Giuliani (University of Parma, Italy). These cell lines were maintained at 37 °C and 5% CO_2_ in RPMI 1640 supplemented with 10% FCS, 2 mM l-glutamine, 100 U/ml penicillin, and 100 U/ml streptomycin (complete medium). The human 293T embryonic kidney cells were purchased from ATCC and were maintained in Dulbecco’s modified Eagle’s supplemented with 10% FCS. All cell lines were mycoplasma-free (EZ-PCR Mycoplasma Test Kit; Biological Industries).

Bone marrow samples from patients with MM were managed at the Division of Hematology, Department of Cellular Biotechnologies and Hematology, University of Rome, Sapienza, Italy (Table 1). Informed consent in accordance with the Declaration of Helsinki was obtained from all patients, and approval was obtained from the ethics committee of the Sapienza University of Rome. The bone marrow aspirates were processed as already described in [68]. In some experiments, myeloma cells were selected using anti-CD138 magnetic beads (Miltenyi Biotec). More than 95% of the purified cells expressed CD138 and CD38.

### Reagents and antibodies

The bromodomain inhibitors JQ1 and I-BET151 (GSK1210151A), OTX015, and C646 (a specific-competitive histone acetyltransferase CBP/EP300 inhibitor) were purchased from Selleckchem.com. The selective inhibitor of the bromodomain-containing transcription factors CREBBP (CBP) and EP300, SGC-CBP30, was purchased from Tocris Bioscience. Lenalidomide was purchased from BioVision Inc. The hetero-bifunctional PROTAC (proteolysis targeting chimera) ARV-825 was purchased from Chemietek. These drugs were dissolved in dimethylsulphoxide (DMSO) and stored at −20 °C until use. The final concentration of DMSO in all experiments was <0.1%. The following mAbs were used for immunostaining or as blocking Abs: anti-MICA (MAB159227), anti-MICB (MAB236511), anti-ULBP-1 (MAB170818), anti-ULBP-2/5/6 (MAB165903), anti-ULBP-3 (MAB166510), and anti-NKG2D (MAB149810) from R&D System, anti-PVR/CD155 (SKII.4) kindly provided by Prof. M. Colonna (Washington University, St. Louis, MO), anti-MHCI (W6/32) provided by Dr. P. Giacomini (Regina Elena Cancer Institute, Rome, Italy), anti-CD56 (C218) mAb provided by Dr. A. Moretta (University of Genoa, Genoa, Italy), APC goat anti-mouse IgG (Poly4053), anti-CD56/PE (HCD56), mouse IgG1/FITC, /PE or /APC isotype control (MOPC-21) purchased from BioLegend. anti-CD3/FITC (SK7), anti-CD56 (NCAM16.2), anti-CD107a/APC (H4A3), anti-CD138-FITC (M15), anti-CD38-APC (HIT2), anti-nectin 2 (R2.525), and anti-CD16-PerCP-Cy5.5 (3G8) purchased from BD Biosciences.

### Flow cytometry and degranulation assay

SKO-007(J3), ARP-1, U266, JJN-3, and RPMI-8226 cells were cultured in six-well tissue culture plates for 72 h at a concentration of 2 × 10^5^ cells/ml in the presence of the indicated drug. The expression of the NKG2D and DNAM-1 ligands on MM cells was analyzed by immunofluorescence staining using unconjugated mAbs, followed by secondary GAM-APC. In all experiments, cells were stained with propidium iodide (PI) (1 μg/ml) in order to assess cell viability (always higher than 90% after the different treatments). Nonspecific fluorescence was assessed by using an isotype-matched irrelevant mAb R&D System, followed by the same secondary antibody. Fluorescence was analyzed using a FACSCalibur flow cytometer (BD Biosciences), and data were analyzed using FlowJo Flow Cytometric Data Analysis Software (Tree Star, Inc). The analysis of ligands expression on patient-derived plasma cells was performed by gating on the CD138^+^ and CD38^+^ PC population.

NK cell-mediated cytotoxicity was evaluated using the lysosomal marker CD107a as previously described [69]. As source of effector cells, we used primary NK cells obtained from PBMCs isolated from healthy donors by Lymphoprep–Nycomed gradient centrifugation and then co-cultured for 10 days with irradiated (30 Gy) Epstein-Barr virus (EBV)-transformed B cell line RPMI 8866 at 37 °C in a humidified 5% CO_2_ atmosphere, as previously described [69]. On day 10, the cell population was routinely more than 90% CD56^+^CD16^+^CD3^−^, as assessed by immunofluorescence and flow cytometry analysis.

When patient-derived plasma cells were used as targets (myeloma cells were selected using anti-CD138 magnetic beads from Miltenyi Biotec), autologous CD138^−^ bone marrow cells were cultured for 2 days in complete medium, supplemented with 200 U/ml IL-2, and used as source of effector cells.

Drug-treated MM cell lines or patient-derived plasma cells were washed twice in complete medium and incubated with NK cells at effector/target (E:T) ratio of 2.5:1, in a U-bottom 96-well tissue culture plate in complete medium at 37 °C and 5% CO_2_ for 2 h. Thereafter, cells were washed with PBS and incubated with anti-CD107a/APC (or cIgG/APC) for 45 min at 4 °C. Cells were then stained with anti-CD3/FITC, anti-CD56/PE, and anti-CD16/PerCP-Cy5.5 to gate the CD3^-^CD56^+^ CD16^+^ NK cell population. In some experiments, cells were pre-treated for 20 min at room temperature with anti-NKG2D neutralizing mAbs or a control Ab (anti-CD56). Fluorescence was analyzed using a FACSCalibur flow cytometer (BD Biosciences), and data were analyzed using FlowJo Flow Cytometric Data Analysis Software (Tree Star).

### Plasmids

MICA-270 bp promoter in pGL3-basic luciferase vector (Promega Corp.) was generated as previously described [68]. To generate the MICA promoter deletions MICA/3.2 kb and MICA/1.2 kb, the appropriate deletion fragments (Kpn-I/HindIII) were generated by PCR according to standard methods from a human 3.2 kb-wild-type-MICA/GFP reporter vector (kindly provided by Dr. Skov, University of Copenhagen, Frederiksberg, Denmark) and cloned in pGL3-basic luciferase vector. The primers used to amplify PCR products using LongAmp *Taq* DNA Polymerase (New England BioLabs) are as follows:

- cagatct**ggtacc**agctcgagaccaacctgaccaac - MICA −3.2 kb-sen;
- cagatct**ggtacc**tggtgggatagggtgaggagatc - MICA −1.2 kb-sen;
- gaatgcc**aagctt**ggccccgacgtcgccaccctctc - MICA +39 bp-rev.

The mutant MICA promoter construct (MICA/-270-DEL) was generated using Quick Change Site-Directed Mutagenesis Kit Statagene, following the manufacturer’s instructions, as described in [13]. All constructs were verified by DNA sequence analysis.

The lentiviral vector pEF.CMV.EGFP-IRF4, encoding the human IRF4, was generated by inserting a full-length human IRF4 complementary DNA (cDNA) (obtained from an expression vector pcDNA3-IRF4, kindly provided by Dr. Hayashi H., Graduate School of Medical Sciences, Nagasaki University, Japan) [70], in the lentivirus pEF.CMV.EGFP. The IRF4 dominant negative expression vector IRF4-DN, encoding a truncated form of the human IRF4, consisting of its N-terminal DNA binding domain (1-405), was generated by inserting the mutant IRF4 cDNA in the pcDNA3 expression vector [70].

The lentiviral copGFP vectors pMIRNA1/pre-miR-125b and the pMIRNA1/control vector were purchased from System Biosciences.

For knocking down cMYC and BRD4, we used the following lentiviral vectors: pLKO.1-sh-cMYC (TRCN0000039642), pLKO.1-sh-BRD4 (TRCN0000382028), and the control vector pLKO non-targeting shRNA MISSION™ (Sigma-Aldrich).

### DNA transfections, virus production, and in vitro transduction

Transfections of SKO-007(J3) cells were carried out using a 4D-Nucleofector System (Lonza). Where needed, cells were transfected in single batches that were then separated into different drug treatment groups, to decrease variations in the experiments due to different transfection efficiency. A pTK-Renilla expression vector was cotransfected to normalize DNA uptake. After 3 h, cells were treated with JQ1; after additional 40 h, cells were harvested and protein extracts were prepared for the luciferase and Renilla assays. Protein concentration was quantified by the BCA method Pierce, Rockford. Luciferase and Renilla activity were quantified using the Dual-Luciferase Reporter Assay and the Glomax Multi Detection System (Promega), following the manufacturer’s instructions.

For lentivirus production, Phoenix cells were transfected with 5 μg of viral DNA using Lipofectamine Plus (Life Technologies). The lentiviral vectors were cotransfected together the packaging vectors pVSVG and psPAX2 into 293T cells using Lipofectamine Plus. After transfection, the cells were placed in fresh medium. After a further 48-h culture, virus-containing supernatants were harvested, filtered, and used immediately for infections. Infections were performed on 0.5 × 10^6^ SKO-007(J3) cells in 2-ml complete medium with polybrene (8 μg/ml) (hexadimethrine bromide; Sigma-Aldrich) for 2 h. For GFP-expressing viruses, the infection efficiency was measured by FACS analysis of GFP expression at day 3 after infection.

### miRNA and mRNA detection, by quantitative real-time polymerase chain reaction (qRT-PCR)

Total RNA was extracted using TRIZOL™ (Life Technologies), according to manufacturer’s instructions. The concentration and quality of the extracted total RNA was determined by measuring light absorbance at 260 nm (A260) and the ratio of A260/A280. Reverse transcription was carried out in a 25-μl reaction volume with 2 μg of total RNA according to the manufacturer’s protocol for M-MLV reverse transcriptase Promega. cDNAs were amplified (TaqMan assays) in triplicate with primers for MICA (Hs00792195_m1), IRF4 (Hs01056533_m1), MYC (Hs00153408_m1), IKZF1 (Hs00958474_m1), IKZF3 (Hs00232635_m1), and GAPDH (Hs03929097_g1) conjugated with fluorochrome FAM (Applied Biosystems). The level of expression was measured using Ct (threshold cycle). The Ct was obtained by subtracting the Ct value of the gene of interest from the housekeeping gene (GAPDH) Ct value. In the present study, we used Ct of the untreated sample as the calibrator. The fold change was calculated according to the formula 2-ΔΔCt, where ΔΔCt was the difference between Ct of the sample and the Ct of the calibrator (according to the formula, the value of the calibrator in each run is 1). The analysis was performed using the SDS version 2.4 software (Applied Biosystems). miRNA quantification was performed using TAQMAN® MICRORNA kit and U6snRNA expression for normalization. Corresponding reverse transcription and polymerase chain reaction primers for U6snRNA and miR-125b were obtained from Applied Biosystems. All PCR reactions were performed using an ABI Prism 7900 Sequence Detection System (Applied Biosystems).

### Western blot analysis

For Western blot analysis, SKO-007(J3) cells were pelleted, washed once with cold phosphate-buffered saline, resuspended in lysis buffer [1% Nonidet P-40 (*v*/*v*), 10% glycerol, 0.1% SDS, 0.5% sodium deoxycholate, 1 mM phenyl-methyl-sulfonyl fluoride (PMSF), 10 mM NaF, 1 mM Na3VO4, complete protease inhibitor mixture Roche in PBS], and subsequently incubated 30 min on ice. The lysate was centrifuged at 14,000 g for 15 min at 4 °C, and the supernatant was collected as whole cell extract. Protein concentration was determined by the BCA method Pierce. Thirty to 50 μg of cell extract was run on 10% denaturing SDS-polyacrylamide gels. Proteins were then electroblotted onto nitrocellulose membranes (Schleicher & Schuell), stained with Ponceau to verify that similar amounts of proteins had been loaded in each lane, and blocked in 5% BSA in TBST buffer. Immunoreactive bands were visualized on the nitrocellulose membranes, using horseradish-peroxidase-coupled goat anti-rabbit or goat anti-mouse immunoglobulins and the ECL detection system (GE Healthcare Amersham), following the manufacturer’s instructions. Antibodies against *β*-actin, IRF4 (H-140), Ikaros (H-100), and Aiolos (L-15) were purchased from Santa Cruz Biotechnology. Antibody against Brd-4 was purchased from Abcam. Antibody against the p85 subunit of PI3 kinase was purchased from Millipore.

### Statistical analysis

Error bars represent SD. Data have been evaluated by paired Student *t* test and a level of *P* < 0.05 was considered to be statistically significant.

## Additional files

Additional file 1: BETi upregulate MICA expression in different human MM cell lines. MICA, MICB, and PVR/CD155 cell surface expression were analyzed by immunofluorescence and flow cytometry on RPMI-8226, U266, ARP-1, and JJN3, respectively, after a 72 h treatment with JQ1. The MFI of MICA, MICB, and PVR/CD155 was calculated based on at least four independent experiments and evaluated by paired Student *t* test (**P* < 0.05). In the insert, a representative histogram of MICA upregulation is shown. The grey-colored histograms represent basal expression of the indicated ligand, while thick black histograms represent the expression after treatment with the drug. (TIF 3542 kb)
Additional file 2: ULBPs and Nec-2 ligand expression on SKO-007(J3) cells following treatment with JQ1. ULBPs and Nec-2 surface expression were analyzed by immunofluorescence and flow cytometry on SKO-007(J3) cells treated with JQ1 as described above for 72 h. The grey-colored histograms represent basal expression of the indicated ligand, while thick black histograms represent the expression after treatment with the drug. Data are representative of three independent experiments. (TIF 3116 kb)
Additional file 3: BETi upregulate MICA mRNA expression in different human MM cell lines. Real-time PCR analysis of total mRNA obtained from the indicated cell lines, untreated or treated with the indicated BETi for 24 and 48 h. Data, expressed as fold change units, were normalized with GAPDH and referred to the untreated cells considered as calibrator and represent the mean of three experiments (**P* < 0.05). (TIF 5669 kb)
Additional file 4: IRF4 and cMYC mRNA expression are inhibited in patient-derived MM PCs upon treatment with BETi. Real-time PCR analysis of total mRNA obtained from purified CD138^+^ cells untreated or treated with I-BET151 for 48 h in complete medium supplemented with 20 ng/ml IL-3 and 2 ng/ml IL-6. Data, expressed as fold change units, were normalized with GAPDH and referred to the untreated cells considered as calibrator. (TIF 4030 kb)
Additional file 5: shRNA interference of cMYC upregulates MICA expression in SKO-007(J3) cells. MICA, MICB and PVR/CD155 cell surface expression were analyzed by flow cytometry on pLKO control (non-targeting) or pLKO-cMYC-lentivirus-infected SKO-007(J3) cells (72 h). (A) Representative histogram of MICA upregulation is shown. Data are representative of one out of three independent experiments. (B) Total RNAs were isolated from infected cells (48 h) for Real-time qRT-PCR analysis. Data, expressed as fold change units, were normalized with GAPDH and referred to the cells infected with non-target shRNA considered as calibrator and represent the mean of three experiments (**P* < 0.05). (TIF 5600 kb)
Additional file 6: (A) Schematic representation of IKAROS and IRF4 response elements of *MICA* and *MICB* promoters. (B, C) Effect of BETi on IKZF1 and IKZF3 mRNA expression in SKO-007(J3) MM cells. Real-time PCR analysis of total mRNA obtained from SKO-007(J3) cells, untreated or treated with JQ1 (0.5 μM) or lenalidomide (5 μM) for 48 h. Data, expressed as fold change units, were normalized with GAPDH and referred to the untreated cells considered as calibrator and represent the mean of three experiments (**P* < 0.05). (D) Lysates of SKO-007(J3) cells untreated or treated with JQ1 or lenalidomide (5 μM) were subjected to Western blotting using anti-IKZF1, anti-IKZF3, and actin antibodies. The proteins transferred to nitrocellulose membranes were stained with Ponceau to verify that similar amounts of proteins had been loaded in each lane. Data are representative of one out of three independent experiments. (E) Real-time PCR analysis of total mRNA obtained from SKO-007(J3) cells, untreated or treated with JQ1, lenalidomide, or the combination of the two drugs as described above for 48 h. Data, expressed as fold change units, were normalized with GAPDH and referred to the untreated cells considered as calibrator and represent the mean of 3 experiments (**P* < 0.05). (F) Lysates of SKO-007(J3) cells untreated or treated with JQ1 and/or lenalidomide (Lena) for 24 or 48 h as described above were subjected to Western blotting using anti-IRF4 and actin antibodies. The proteins transferred to nitrocellulose membranes were stained with Ponceau to verify that similar amounts of proteins had been loaded in each lane. Data are representative of one out of two independent experiments. Densitometric analysis of normalized IRF4/actin is shown. (TIF 8785 kb)
Additional file 7: (A, B) Treatment with the indicated concentrations of JQ1 (48 h) represses IRF4 and upregulates MICA mRNA expression in SKO-007(J3) cells. Data of real-time PCR analysis, expressed as fold change units, were normalized with GAPDH and referred to the untreated cells considered as calibrator. Data represent the mean of three experiments (**P* < 0.05). (TIF 2956 kb)
Additional file 8: Schematic representation of the PROTAC ARV-825. (TIF 3981 kb)
Additional file 9: shRNA interference of BRD4 upregulates MICA expression in SKO-007(J3) cells. (A) MICA, MICB, and PVR/CD155 cell surface expression were analyzed by flow cytometry on pLKO-control (non-targeting) or pLKO-BRD4-lentivirus-infected SKO-007(J3) cells (72 h). The MFI of MICA, MICB, and PVR/CD155 was calculated based on three independent experiments and evaluated by paired Student *t* test (**P* < 0.05). (B) A representative histogram of MICA upregulation is shown. (TIF 2314 kb)
Additional file 10: MICA cell surface expression was analyzed by flow cytometry on SKO-007(J3) cells treated with OTX015 (0.5 μM) for 72 h. The MFI of MICA were calculated based on at least four independent experiments and evaluated by paired Student *t* test (**P* < 0.05). The white-colored histogram represents basal expression of MICA, while the grey histogram represents the expression after treatment with the drug. (TIF 2407 kb)

## Abbreviations

AML: Acute myeloid leukemia
CBP: Cyclic AMP response element binding protein (CREB) binding protein
ChIP: Chromatin immunoprecipitation assay
CLR4: Cullin-4-RING ubiquitin ligase complex
CRBN: Cereblon
CUL4: Cullin-4
DDB1: DNA damage binding protein-1
DDR: DNA damage response
DDB: DNA binding domain
EP300: E1A interacting protein of 300 kDa
HDAC: Histone deacetylase
IMiDs: Immunomodulatory drugs
MDS: Myelodysplastic syndrome
MGUS: Monoclonal gammopathy of undetermined significance
Nec-2: Nectin 2
NK: Natural killer
PBMC: Peripheral blood mononuclear cells
PROTAC: Proteolysis targeting chimera
PVR: Poliovirus receptor
ROC1: RING finger protein
shRNA: Short hairpin RNA
SMM: Smoldering multiple myeloma
ULBP: UL16 binding proteins
