# Supplementary figures and images for: Inhibition of bromodomain and extra-terminal (BET) proteins increases NKG2D ligand MICA expression and sensitivity to NK cell-mediated cytotoxicity in multiple myeloma cells: role of cMYC-IRF4-miR-125b interplay
Source: J Hematol Oncol. 2016 Dec 1;9:134. doi: 10.1186/s13045-016-0362-2 (PMC5131470; doi:10.1186/s13045-016-0362-2)

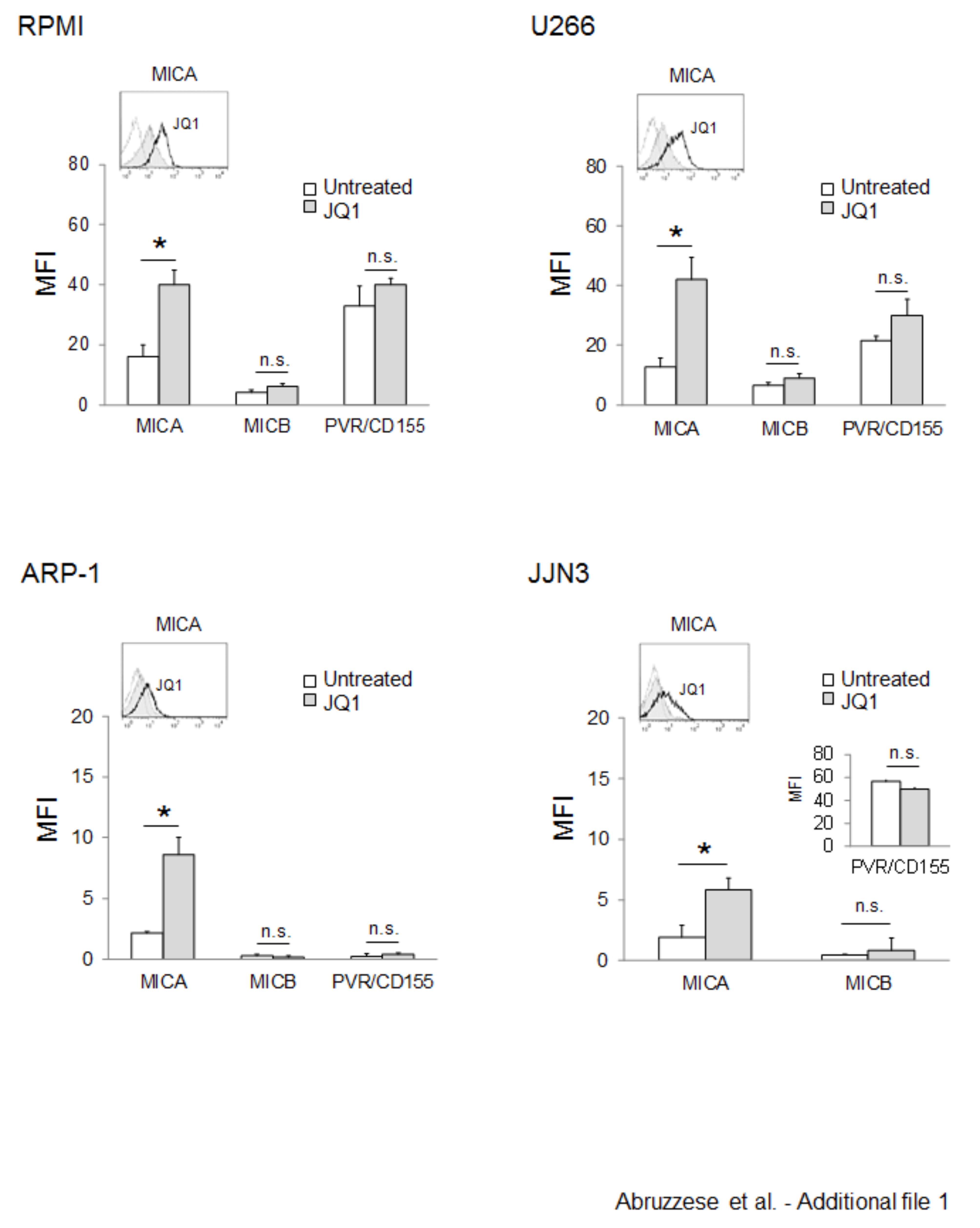

Supplement: Additional file 1: — BETi upregulate MICA expression in different human MM cell lines. MICA, MICB, and PVR/CD155 cell surface expression were analyzed by immunofluorescence and flow cytometry on RPMI-8226, U266, ARP-1, and JJN3, respectively, after a 72 h treatment with JQ1. The MFI of MICA, MICB, and PVR/CD155 was calculated based on at least four independent experiments and evaluated by paired Student t test (*P < 0.05). In the insert, a representative histogram of MICA upregulation is shown. The grey-colored histograms represent basal expression of the indicated ligand, while thick black histograms represent the expression after treatment with the drug. (TIF 3542 kb) [file 13045_2016_362_MOESM1_ESM.tif]

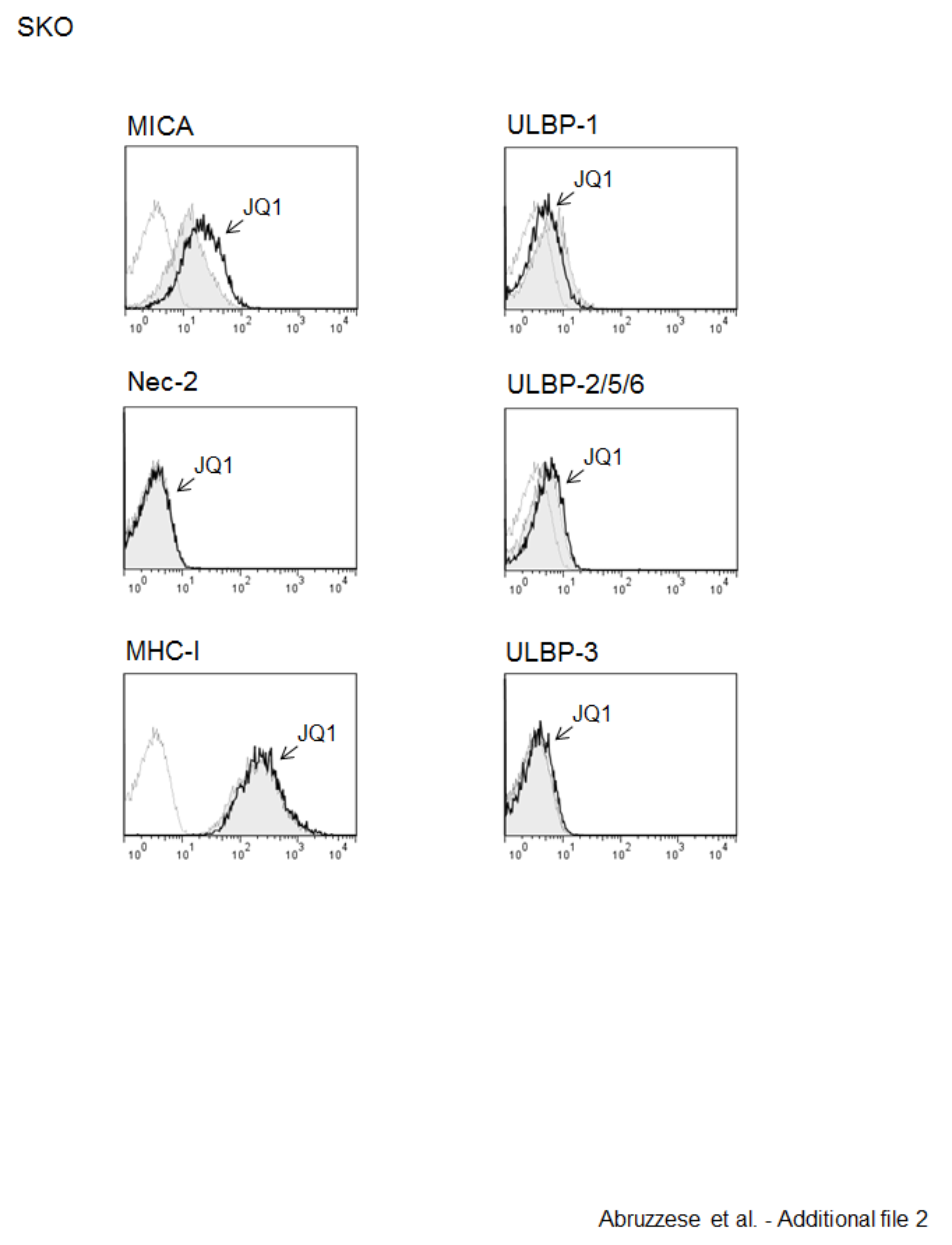

Supplement: Additional file 2: — ULBPs and Nec-2 ligand expression on SKO-007(J3) cells following treatment with JQ1. ULBPs and Nec-2 surface expression were analyzed by immunofluorescence and flow cytometry on SKO-007(J3) cells treated with JQ1 as described above for 72 h. The grey-colored histograms represent basal expression of the indicated ligand, while thick black histograms represent the expression after treatment with the drug. Data are representative of three independent experiments. (TIF 3116 kb) [file 13045_2016_362_MOESM2_ESM.tif]

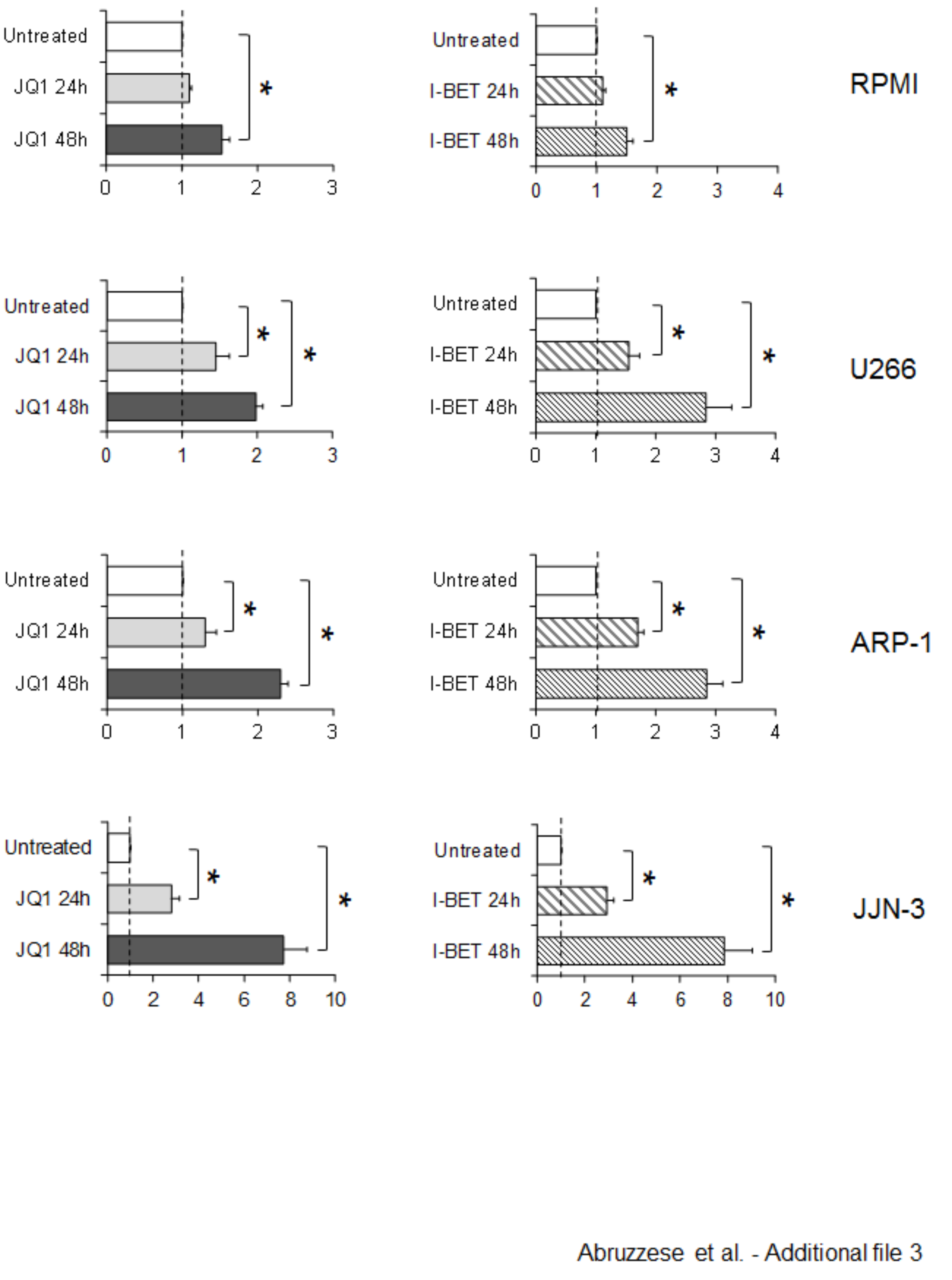

Supplement: Additional file 3: — BETi upregulate MICA mRNA expression in different human MM cell lines. Real-time PCR analysis of total mRNA obtained from the indicated cell lines, untreated or treated with the indicated BETi for 24 and 48 h. Data, expressed as fold change units, were normalized with GAPDH and referred to the untreated cells considered as calibrator and represent the mean of three experiments (*P < 0.05). (TIF 5669 kb) [file 13045_2016_362_MOESM3_ESM.tif]

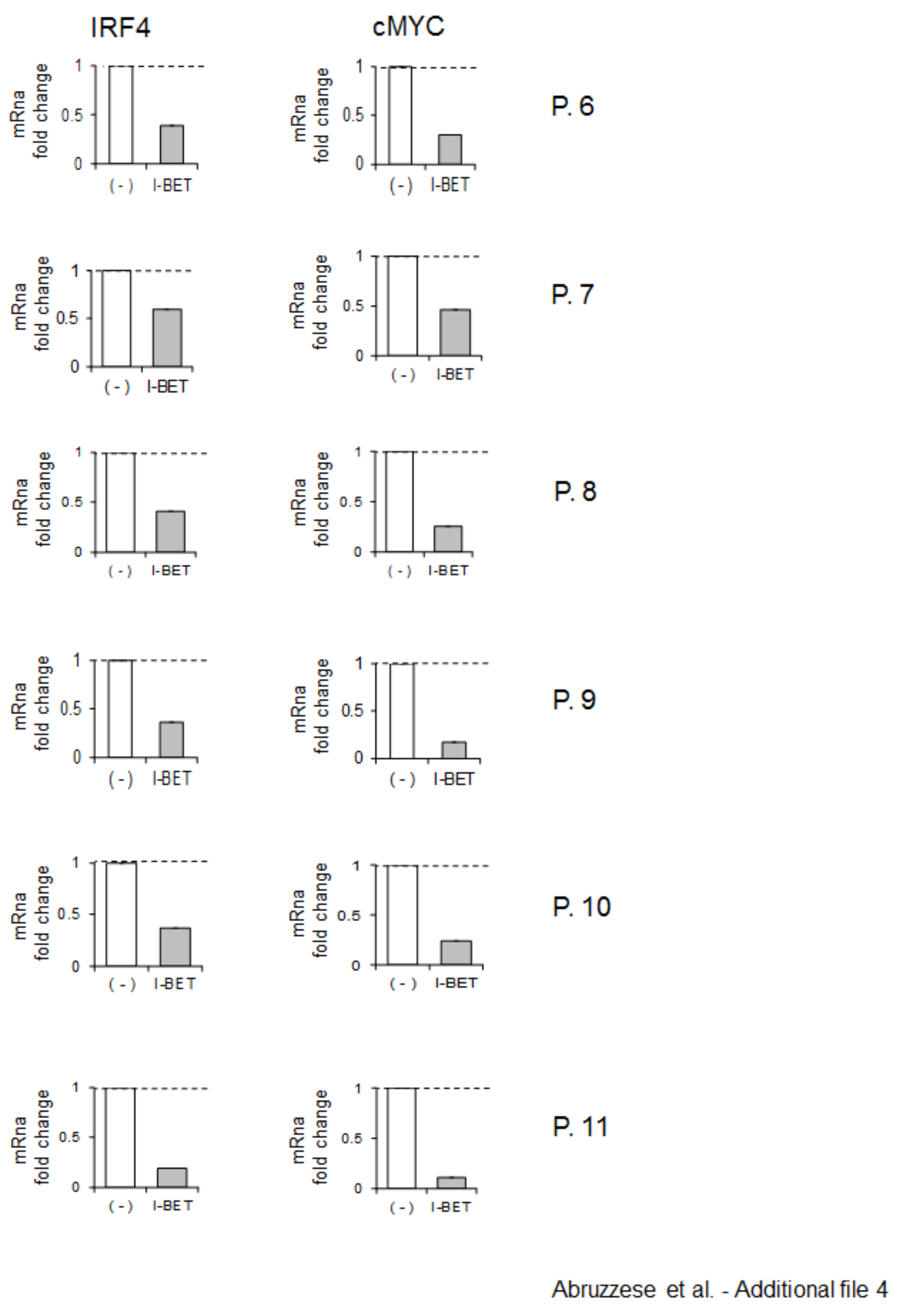

Supplement: Additional file 4: — IRF4 and cMYC mRNA expression are inhibited in patient-derived MM PCs upon treatment with BETi. Real-time PCR analysis of total mRNA obtained from purified CD138+ cells untreated or treated with I-BET151 for 48 h in complete medium supplemented with 20 ng/ml IL-3 and 2 ng/ml IL-6. Data, expressed as fold change units, were normalized with GAPDH and referred to the untreated cells considered as calibrator. (TIF 4030 kb) [file 13045_2016_362_MOESM4_ESM.tif]

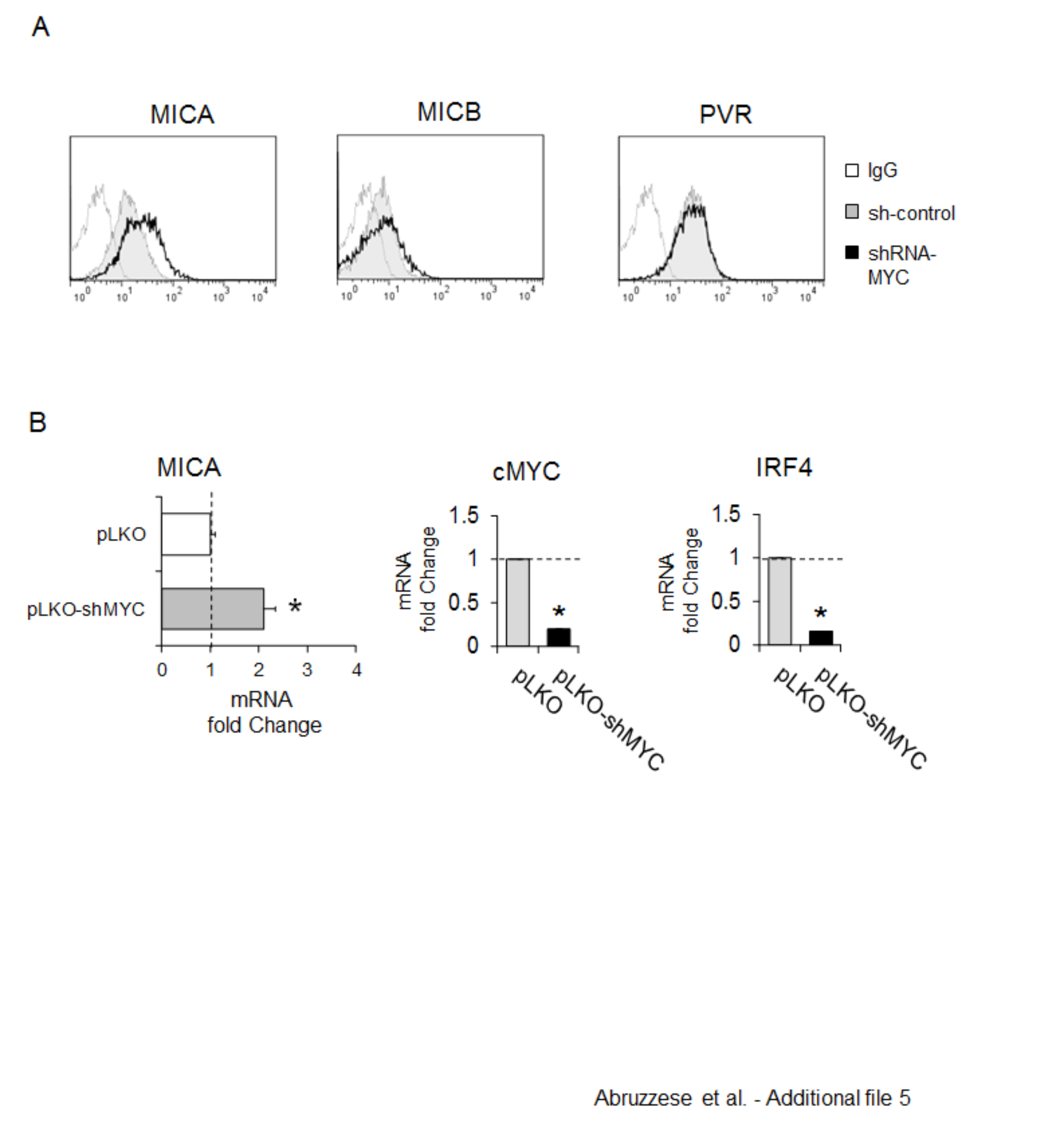

Supplement: Additional file 5: — shRNA interference of cMYC upregulates MICA expression in SKO-007(J3) cells. MICA, MICB and PVR/CD155 cell surface expression were analyzed by flow cytometry on pLKO control (non-targeting) or pLKO-cMYC-lentivirus-infected SKO-007(J3) cells (72 h). (A) Representative histogram of MICA upregulation is shown. Data are representative of one out of three independent experiments. (B) Total RNAs were isolated from infected cells (48 h) for Real-time qRT-PCR analysis. Data, expressed as fold change units, were normalized with GAPDH and referred to the cells infected with non-target shRNA considered as calibrator and represent the mean of three experiments (*P < 0.05). (TIF 5600 kb) [file 13045_2016_362_MOESM5_ESM.tif]

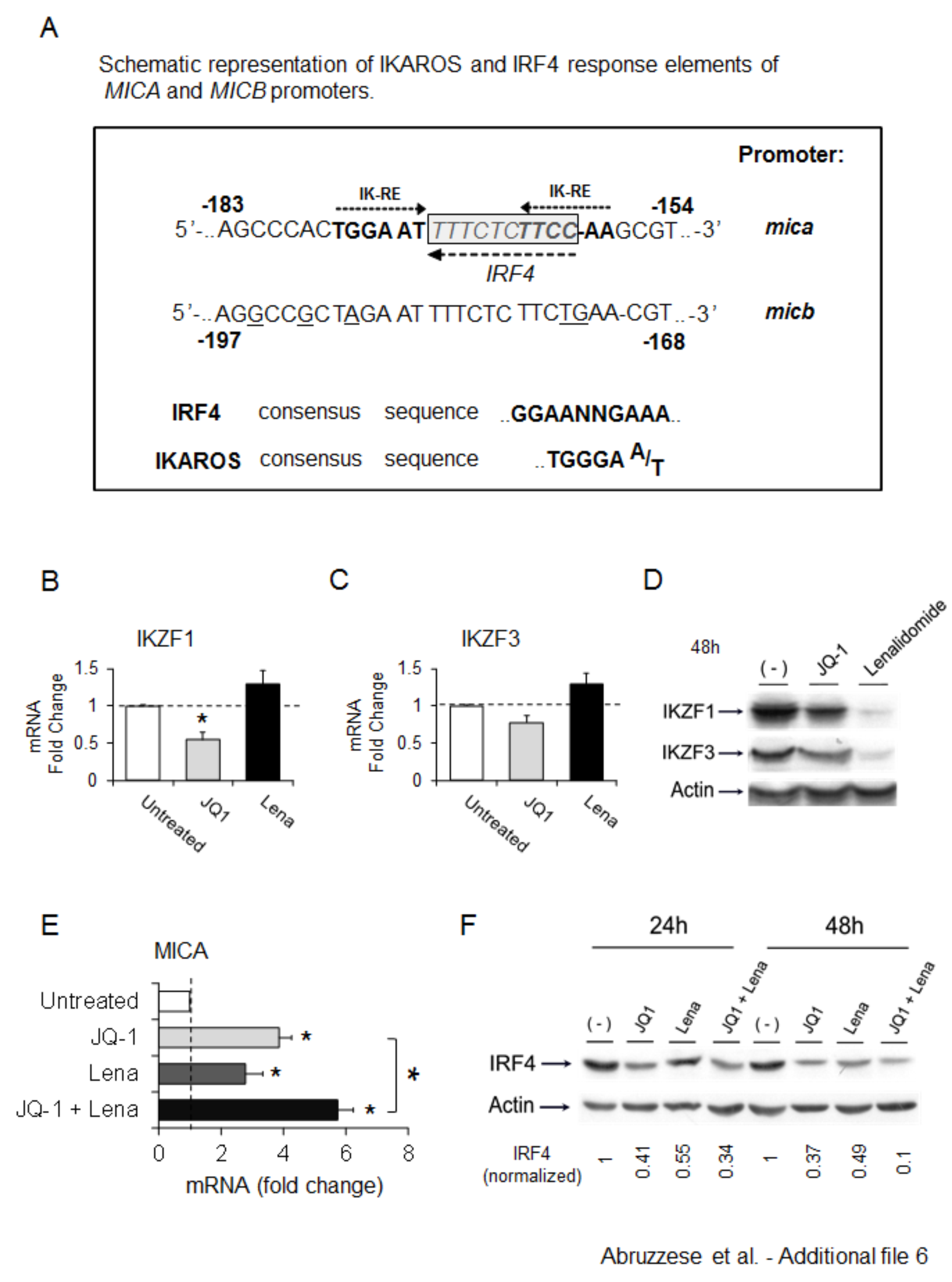

Supplement: Additional file 6: — (A) Schematic representation of IKAROS and IRF4 response elements of MICA and MICB promoters. (B, C) Effect of BETi on IKZF1 and IKZF3 mRNA expression in SKO-007(J3) MM cells. Real-time PCR analysis of total mRNA obtained from SKO-007(J3) cells, untreated or treated with JQ1 (0.5 μM) or lenalidomide (5 μM) for 48 h. Data, expressed as fold change units, were normalized with GAPDH and referred to the untreated cells considered as calibrator and represent the mean of three experiments (*P < 0.05). (D) Lysates of SKO-007(J3) cells untreated or treated with JQ1 or lenalidomide (5 μM) were subjected to Western blotting using anti-IKZF1, anti-IKZF3, and actin antibodies. The proteins transferred to nitrocellulose membranes were stained with Ponceau to verify that similar amounts of proteins had been loaded in each lane. Data are representative of one out of three independent experiments. (E) Real-time PCR analysis of total mRNA obtained from SKO-007(J3) cells, untreated or treated with JQ1, lenalidomide, or the combination of the two drugs as described above for 48 h. Data, expressed as fold change units, were normalized with GAPDH and referred to the untreated cells considered as calibrator and represent the mean of 3 experiments (*P < 0.05). (F) Lysates of SKO-007(J3) cells untreated or treated with JQ1 and/or lenalidomide (Lena) for 24 or 48 h as described above were subjected to Western blotting using anti-IRF4 and actin antibodies. The proteins transferred to nitrocellulose membranes were stained with Ponceau to verify that similar amounts of proteins had been loaded in each lane. Data are representative of one out of two independent experiments. Densitometric analysis of normalized IRF4/actin is shown. (TIF 8785 kb) [file 13045_2016_362_MOESM6_ESM.tif]

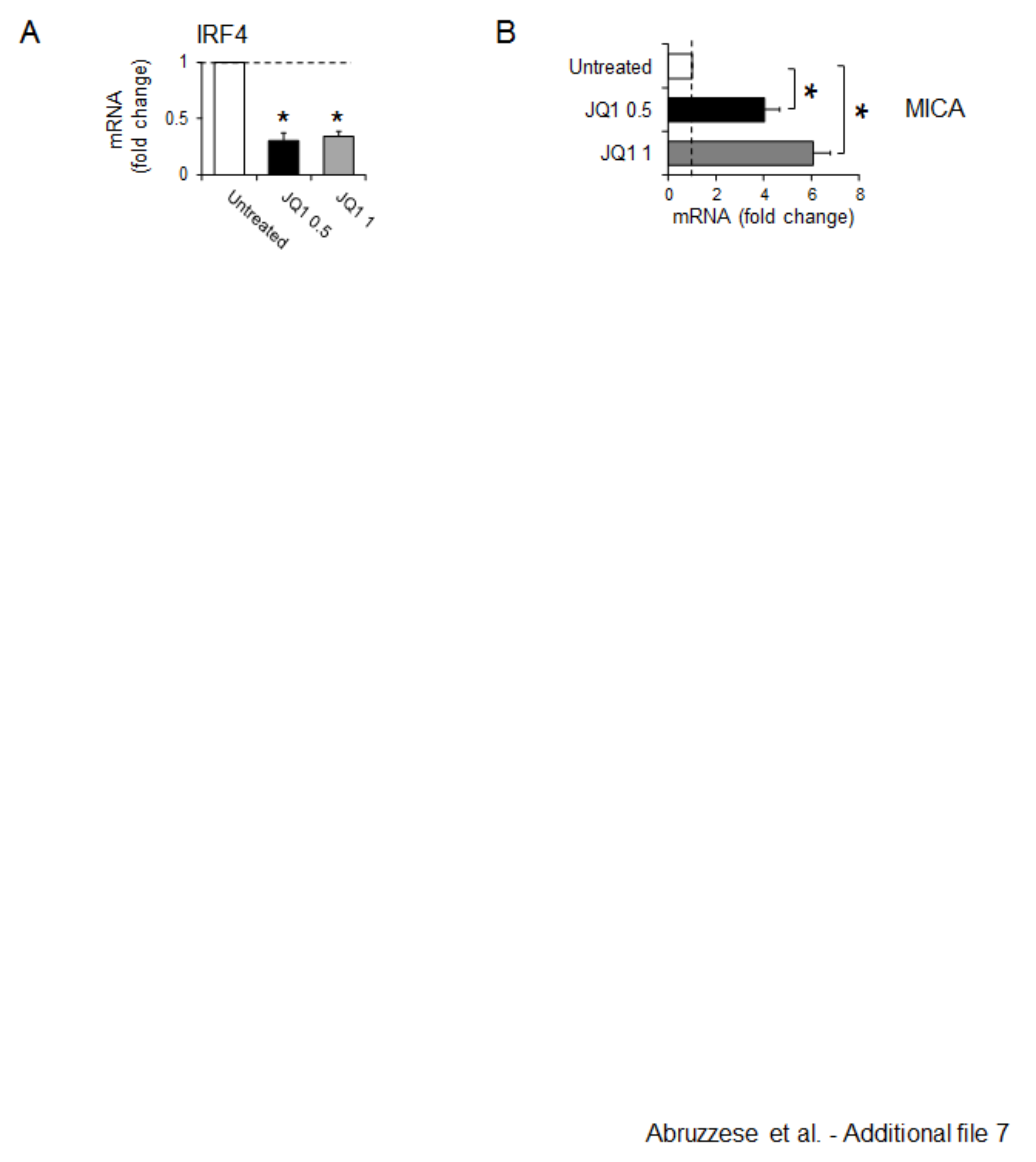

Supplement: Additional file 7: — (A, B) Treatment with the indicated concentrations of JQ1 (48 h) represses IRF4 and upregulates MICA mRNA expression in SKO-007(J3) cells. Data of real-time PCR analysis, expressed as fold change units, were normalized with GAPDH and referred to the untreated cells considered as calibrator. Data represent the mean of three experiments (*P < 0.05). (TIF 2956 kb) [file 13045_2016_362_MOESM7_ESM.tif]

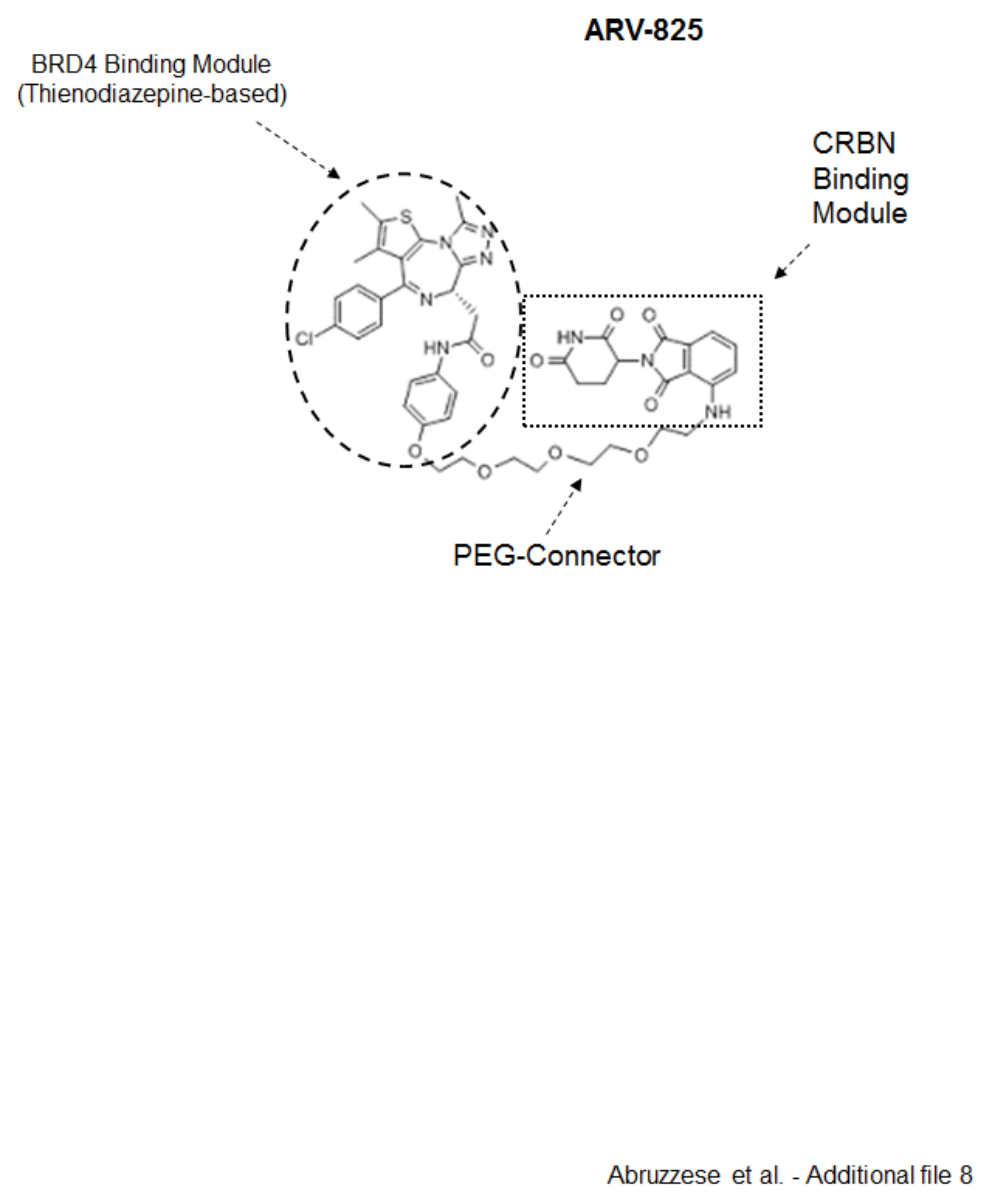

Supplement: Additional file 8: — Schematic representation of the PROTAC ARV-825. (TIF 3981 kb) [file 13045_2016_362_MOESM8_ESM.tif]

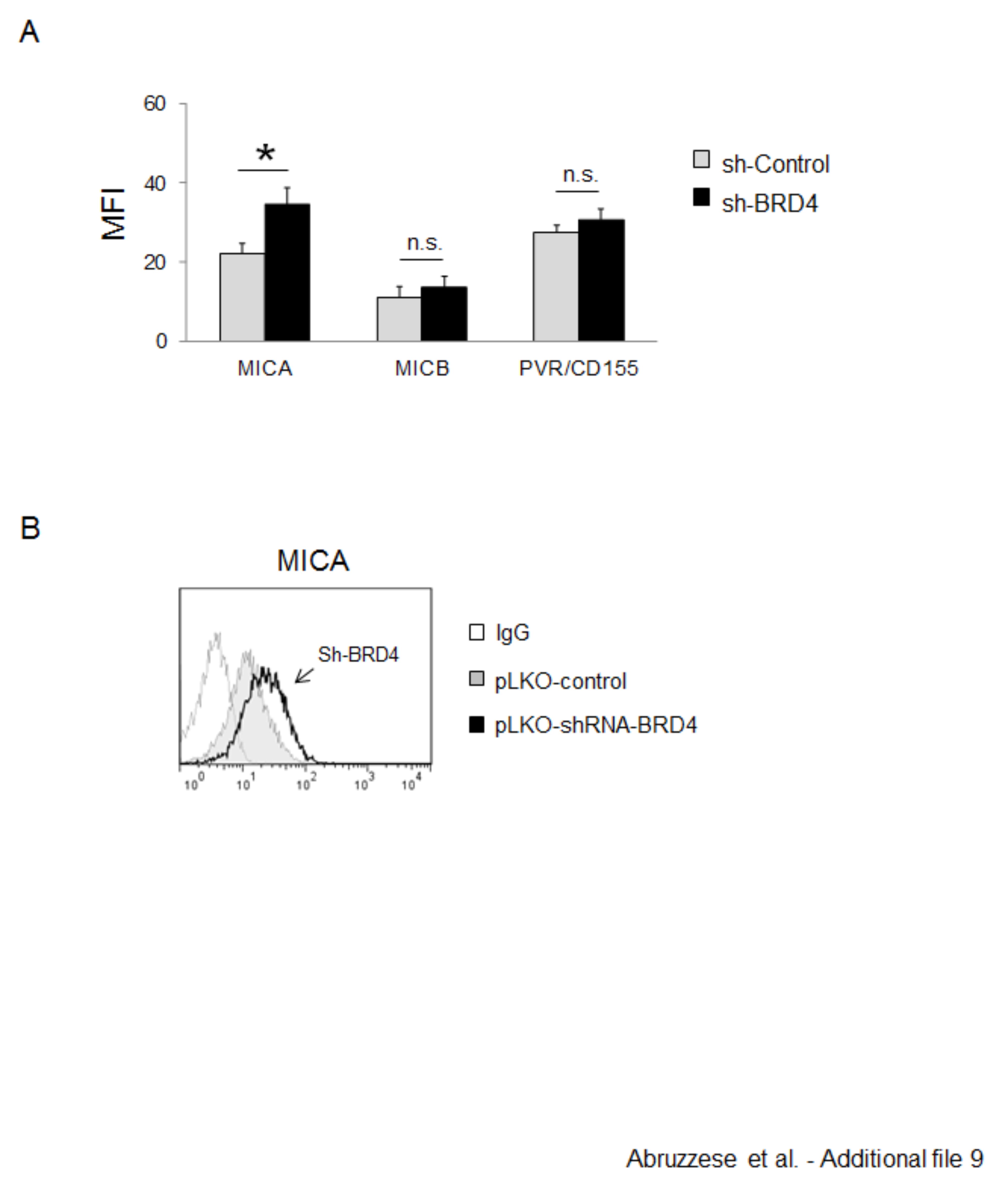

Supplement: Additional file 9: — shRNA interference of BRD4 upregulates MICA expression in SKO-007(J3) cells. (A) MICA, MICB, and PVR/CD155 cell surface expression were analyzed by flow cytometry on pLKO-control (non-targeting) or pLKO-BRD4-lentivirus-infected SKO-007(J3) cells (72 h). The MFI of MICA, MICB, and PVR/CD155 was calculated based on three independent experiments and evaluated by paired Student t test (*P < 0.05). (B) A representative histogram of MICA upregulation is shown. (TIF 2314 kb) [file 13045_2016_362_MOESM9_ESM.tif]

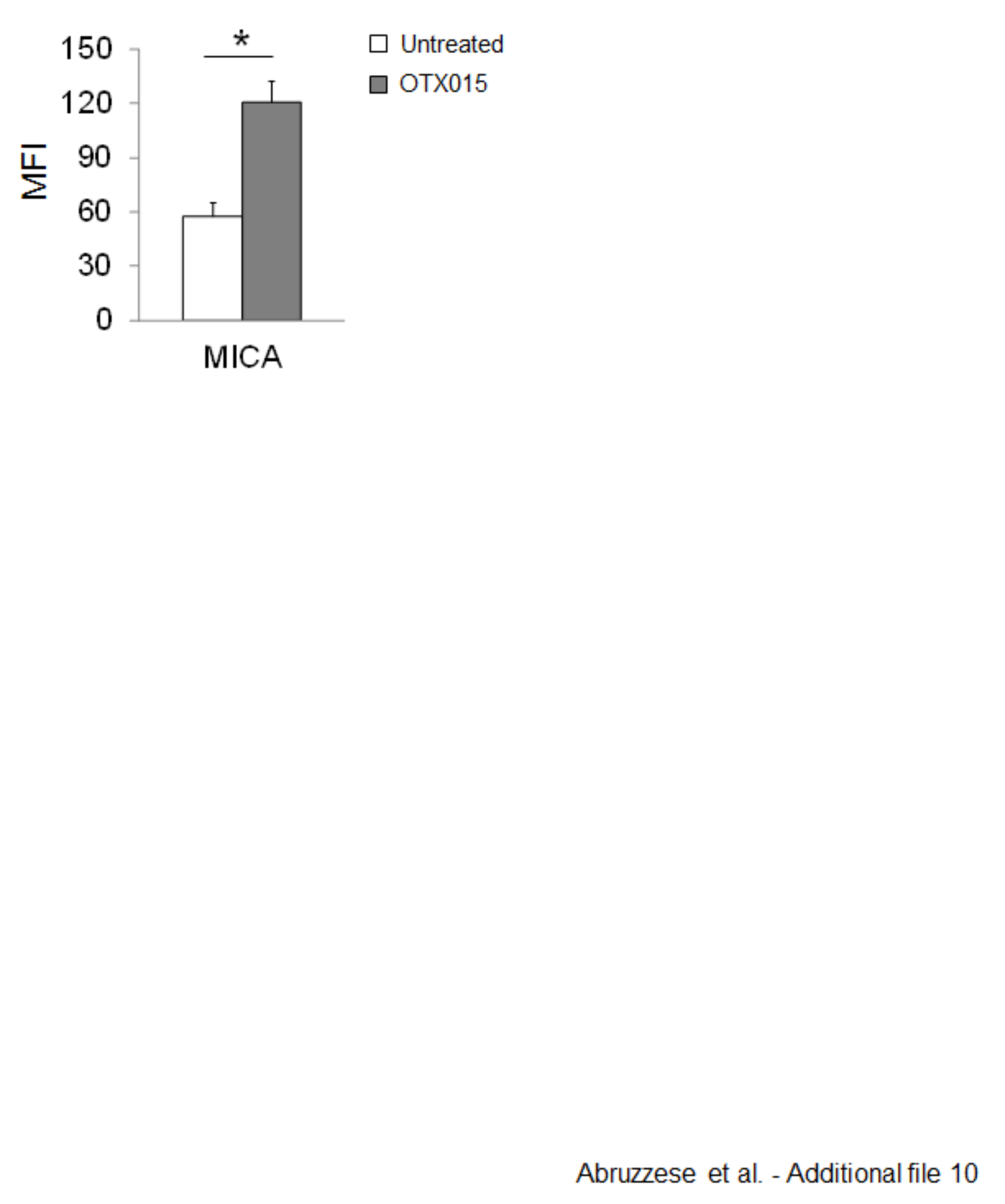

Supplement: Additional file 10: — MICA cell surface expression was analyzed by flow cytometry on SKO-007(J3) cells treated with OTX015 (0.5 μM) for 72 h. The MFI of MICA were calculated based on at least four independent experiments and evaluated by paired Student t test (*P < 0.05). The white-colored histogram represents basal expression of MICA, while the grey histogram represents the expression after treatment with the drug. (TIF 2407 kb) [file 13045_2016_362_MOESM10_ESM.tif]
